# Task-Related Controllability of Functional Connectome During a Working Memory Task in Schizophrenia, Bipolar Disorder, and Major Depressive Disorder

**DOI:** 10.34133/research.0792

**Published:** 2025-08-05

**Authors:** Jun Yang, Zhening Liu, Feiwen Wang, Wenjian Tan, Danqing Huang, Xuan Ouyang, Haojuan Tao, Guowei Wu, Yunzhi Pan, Jie Yang, Lena Palaniyappan

**Affiliations:** ^1^ Department of Psychiatry, and National Clinical Research Center for Mental Disorders, The Second Xiangya Hospital of Central South University, Changsha, Hunan, China.; ^2^ Douglas Mental Health University Institute, Department of Psychiatry, McGill University, Montreal, Quebec, Canada.; ^3^ Department of Psychiatry, Schulich School of Medicine and Dentistry, Western University, London, Ontario, Canada.; ^4^ Robarts Research Institute, Schulich School of Medicine and Dentistry, Western University, London, Ontario, Canada.

## Abstract

Working memory (WM) deficit is a prominent and common cognitive impairment in major psychiatric disorders (MPDs). Altered control of brain state transitions may underlie the neural basis of WM deficit. We investigate whether shared and illness-specific alterations in controllability underlie WM deficits in MPDs. We examined functional magnetic resonance imaging data during an *n*-back WM task from 105 patients with schizophrenia (SZ), 67 with bipolar disorder (BD), 51 with major depressive disorder (MDD), and 80 healthy controls (HCs). We calculated each brain region’s capacity to steer transitions to connectomic states with less input (average controllability) and to difficult-to-reach states with high input (modal controllability). The effect of altered controllability on clinical and cognitive characteristics and their likely genetic and neurotransmitter basis were investigated. All MPDs demonstrated a common but graded pattern of reduced modal controllability within the frontoparietal network compared to HC, with SZ showing the most pronounced impairment. Relative to BD and MDD, SZ exhibited the broadest profile of reduced average and modal controllability across the cortex, particularly in sensory, default mode, and salience networks. The affected brain regions preferentially expressed genes that determine synaptic biology and chemoarchitecture involving glutamate/γ-aminobutyric acid (GABA) and monoamine [dopamine and 5-hydroxytryptamine (5-HT)] neurotransmitter systems. A graded, transdiagnostic reduction in the influence of the sensory networks and triple network system in implementing state transitions underlies WM deficits in MPDs. This deficit, especially pronounced in SZ, has its likely basis in synaptic biology and in glutamate/GABA and monoamine (dopamine and 5-HT) neurotransmitters.

## Introduction

Working memory (WM) refers to the ability to store, manipulate, and update temporary information for goal-directed behavior, which is the basis of advanced cognitive functions [1]. WM deficits have long been recognized as common cognitive impairments in major psychiatric disorders [MPDs; e.g., schizophrenia (SZ), bipolar disorders (BDs), and major depressive disorders (MDDs)], persisting even after remission [2–4] and markedly affecting patients’ quality of life and functional outcomes [5,6]. These deficits are most severe in SZ, followed by BD and MDD [7]. While convergent studies have implicated that MPDs share overlapping, graded, and distinct task-evoked abnormal activation of brain regions and molecular substrates associated with WM [8–11], the transdiagnostic neurobiological mechanisms underlying WM deficits still need to be unraveled.

To address this gap, we focus on impaired brain dynamics—specifically, the failure of adaptive network reconfiguration required for WM. WM tasks require dynamic brain state transitions achieved through adaptive reconfiguration and modulation of network components [e.g., frontoparietal network (FPN) and default mode network (DMN)], which enable optimal information processing and maintenance [12–14]. Our previous work reported functional reconfiguration in SZ, characterized by load-dependent increased instability of distributed connectivity of the supplementary motor area [15]. BD and SZ shared disrupted dynamic modulation within DMN under WM tasks [16]. Together, these findings implicate a transdiagnostic impairment in adaptive network reconfiguration and modulation across MPDs. This dysfunction may hinder efficient brain state transitions, providing a unified mechanistic basis for WM deficits. However, the computational principles governing these failures remain uncharacterized.

Network control theory, originating from engineering, provides a framework to examine brain dynamics and manipulate network components, aiming to achieve a desirable functional state [17,18]. Of the various metrics of network control, average controllability refers to the average energy needed to steer the system into different states with little effort, which quantifies the ability of areas that can push the brain to easy-to-reach states [17]. Modal controllability refers to the capacity to control the specific patterns of brain state transitions over time [19], which quantifies the ability of areas that can move the brain into difficult-to-reach states [17]. In addition, a comparison between structural and functional controllability metrics is elaborated in File S1.

Brain controllability during resting state has been previously reported in MPDs. These studies have highlighted the presence of disrupted average and modal controllability across the triple network system and sensorimotor network in relation to symptom burden in first-episode never-treated SZ [20] and in BD [21]. Disrupted resting-state controllability in MDD appears to carry information that can potentially inform treatment choices (antidepressants/physical therapies) [22,23]. We do not yet know how complex task processing demands, which place greater energy cost and involve difficult-to-reach functional states [24], constrain state transitions in MPDs.

**Fig. 1.**
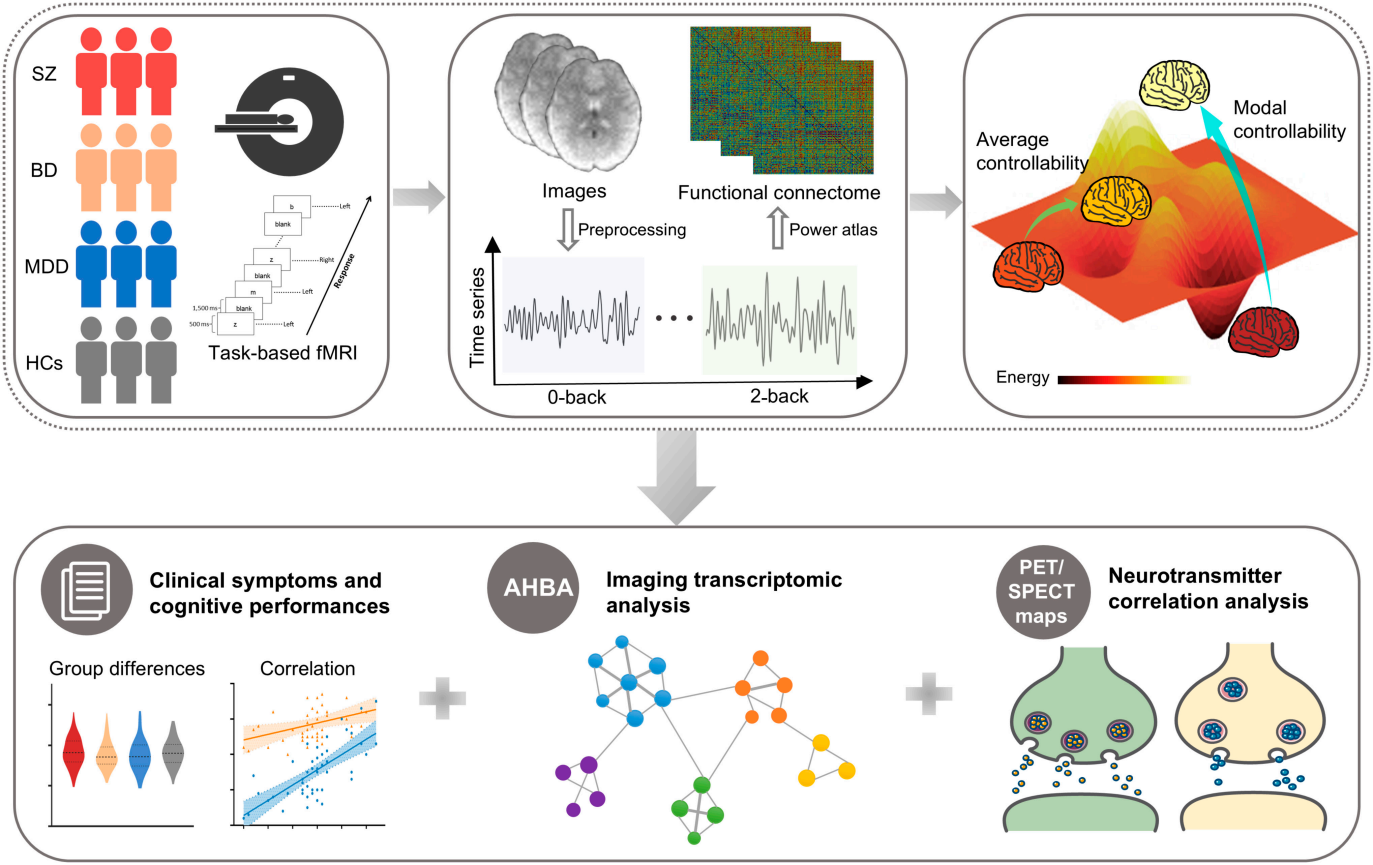
A schematic overview of the study design and analysis pipeline. This study collected *n*-back task-based fMRI data from a transdiagnostic sample of patients with SZ, BD, MDD, and HCs. The time series under 0-back and 2-back were extracted and functional connectomes based on Power atlas [60] were constructed. Controllability metrics were calculated in the functional matrices. Average controllability quantifies the ability to transition into easy-to-reach states with little effort; modal controllability quantifies the ability to transition into difficult-to-reach states with large effort. We further identified the difference in controllability among groups and explored associations between abnormal controllability and clinical symptoms, cognitive performances, gene expression, and neurotransmitter profiles.

This study examined brain network controllability based on the *n*-back WM functional magnetic resonance imaging (fMRI) data from a transdiagnostic sample including patients with SZ, BD, and MDD and healthy controls (HCs). We aimed to characterize brain controllability patterns across different WM loads and their overlapping features across 3 patient groups. We related disrupted controllability to symptom burden and WM performance. Moreover, recent development of brain-wide gene expression atlases [25] and positron emission tomography (PET)/single-photon emission computed tomography (SPECT)-derived neurotransmitter receptor/transporter maps [26] has provided opportunities to bridge the gap between brain function and its molecular substrates. By integrating neuroimaging with gene and neurotransmitter data, studies have revealed how disease-related disruptions in microscale architecture drive macroscale brain abnormalities [27–30]. We indirectly explored the molecular mechanisms underpinning the identified network controllability by analyzing spatial correlations with known patterns of gene expression and the chemoarchitecture of the brain. A schematic overview of the study design and analysis pipeline is shown in Fig. 1.

## Results

### Demographic and clinical characteristics

A total of 303 participants (105 patients with SZ, 67 patients with BD, 51 patients with MDD, and 80 HCs) were enrolled in this study. The demographic and clinical characteristics are shown in Table. There were significant differences in age and education years across 4 groups and illness duration and medication across patient groups. SZ showed higher a brief psychiatric rating scale (BPRS) score than BD and MDD. BD had higher Young mania rating scale (YMRS) scores and lower Hamilton rating scale for depression (HAMD) and Hamilton rating scale for anxiety (HAMA) scores than MDD. Patient groups all exhibited poorer WM task performances, including lower “0-back” and “2-back” reaction times and target accuracy, than HCs. SZ had the lowest 0-back and 2-back target accuracies among 4 groups. Among BD, there were 29 patients in a depressive state, 13 in a manic/hypomanic state, 2 in a mixed state, and 23 in a euthymic state.

**Table.** Demographic and clinical characteristics of each group

Variables	SZ (*n* = 105)	BD (*n* = 67)	MDD (*n* = 51)	HCs (*n* = 80)	χ^2^/*F*/*t*	*P*	Post hoc analysis	
							SZ/BD/MDD vs. HCs	SZ vs. BD vs. MDD
Gender (M/F)	63/42	30/37	28/23	39/41	4.53 ^a^	0.209	N/A	N/A
Age (years)	25.32 ± 5.59	26.19 ± 5.65	29.45 ± 8.12	23.23 ± 4.30	12.21 ^b^	<0.001	SZ > HCs^*^	SZ < MDD^***^
							BD > HCs^**^	BD < MDD^**^
							MDD > HCs^***^	
Education (years)	11.87 ± 2.74	13.31 ± 2.79	12.14 ± 3.07	13.95 ± 2.56	10.36 ^b^	<0.001	SZ < HCs^***^	SZ < BD^**^
							MDD < HCs^***^	MDD < BD ^*^
Illness duration (m)	28.25 ± 32.01	52.32 ± 51.45	47.56 ± 58.76	–	6.67 ^b^	0.002	N/A	SZ < BD^**^
							N/A	SZ < MDD^*^
CPZ	410.10 ± 211.32	227.98 ± 247.02	54.72 ± 150.31	–	51.00 ^b^	<0.001	N/A	SZ > BD^***^
							N/A	SZ > MDD^***^
							N/A	BD > MDD^***^
FLU	0.80 ± 4.06	9.97 ± 14.45	23.63 ± 18.25	–	61.77 ^b^	<0.001	N/A	SZ < BD^***^
							N/A	SZ < MDD^***^
							N/A	BD < MDD^***^
SANS	36.57 ± 28.54	–	–	–	–	–	N/A	N/A
SAPS	20.86 ± 15.41	–	–	–	–	–	N/A	N/A
BPRS	37.97 ± 11.30	26.78 ± 7.69	28.69 ± 5.78	–	28.76 ^b^	<0.001	N/A	SZ > BD^***^
							N/A	SZ > MDD^***^
YMRS	–	6.57 ± 8.78	2.35 ± 2.74	–	3.65 ^c^	<0.001	N/A	N/A
HAMD	–	12.78 ± 9.61	19.41 ± 6.38	–	-4.45 ^c^	<0.001	N/A	N/A
HAMA	–	9.97 ± 9.33	15.55 ± 8.51	–	-3.32	0.001	N/A	N/A
0-back target ACC	0.73 ± 0.28	0.88 ± 0.15	0.90 ± 0.15	0.92 ± 0.14	14.09 ^b^	<0.001	SZ < HCs^***^	SZ < BD^***^
								SZ < MDD^***^
0-back target RT	559.18 ± 151.85	531.23 ± 97.95	540.39 ± 133.38	481.85 ± 90.59	5.57 ^b^	0.001	SZ > HCs^***^	
							BD > HCs^*^	
							MDD > HCs^*^	
2-back target ACC	0.49 ± 0.25	0.60 ± 0.27	0.62 ± 0.23	0.74 ± 0.18	16.19 ^b^	<0.001	SZ < HCs^***^	SZ < BD^**^
							BD < HCs^***^	SZ < MDD^**^
							MDD < HCs^*^	
2-back target RT	701.51 ± 173.90	738.57 ± 173.76	716.44 ± 225.60	642.04 ± 139.57	3.49 ^b^	0.016	SZ > HCs^*^	
							BD > HCs^**^	
							MDD > HCs^*^	

Note: Quantitative data were presented as means ± SD. M/F, male/female; m, month; CPZ, chlorpromazine equivalent dose [64]; FLU, fluoxetine equivalents dose [65].

^a^ χ^2^ test.

^b^ One-way ANOVA.

^c^ Two-sample *t* test.

^*^P < 0.05; ^**^P < 0.01; ^***^P < 0.001.

### Group differences in controllability metrics

**Fig. 2.**
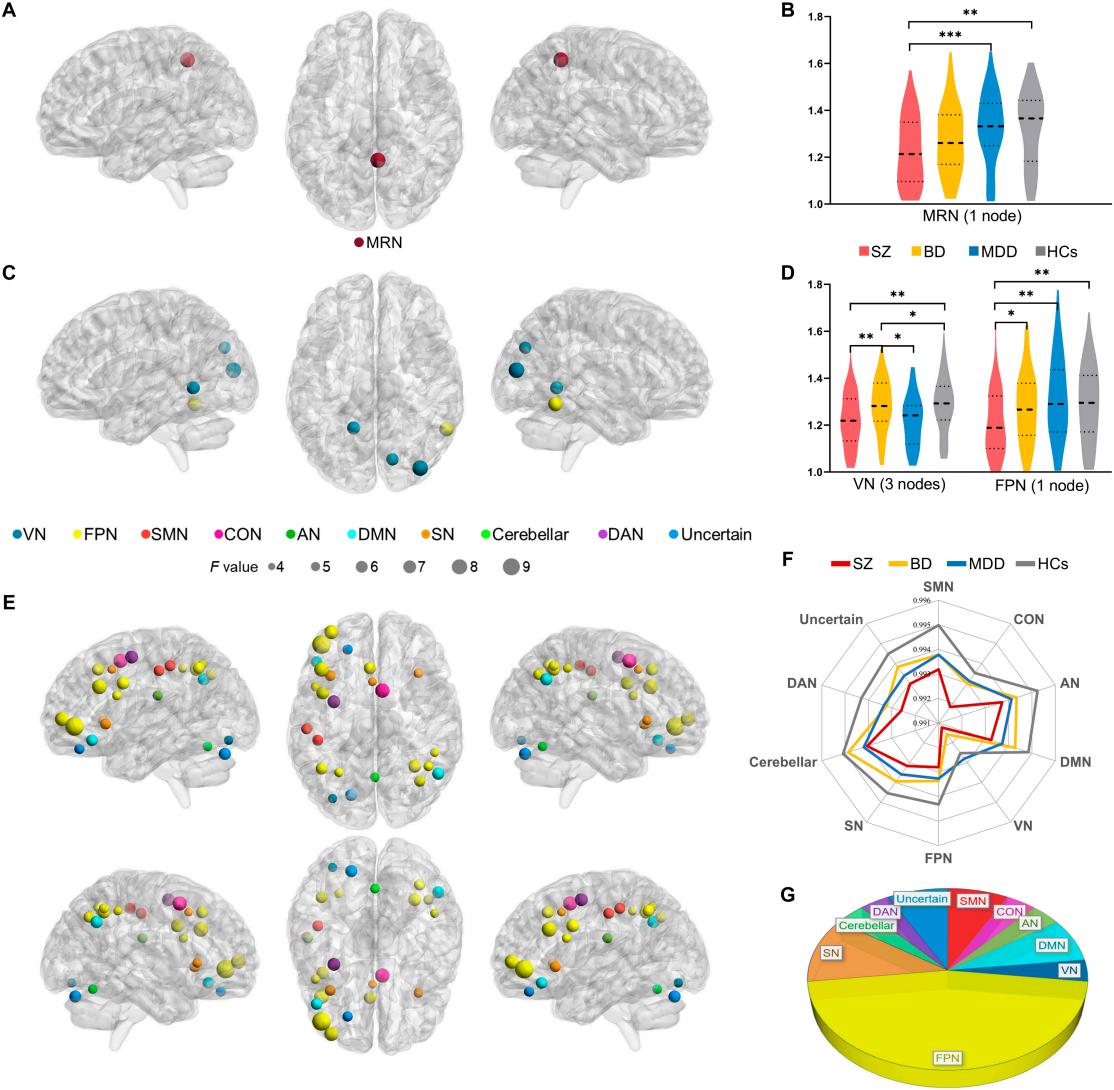
Brain regions with significant differences in average and modal controllability. (A and C) Brain maps show the regions with omnibus differences in average controllability under 0-back and 2-back loads, respectively. (B and D) Violin plots show average controllability values in detected regions for each group. In (D), the mean values of average controllability of 3 nodes in VN are adopted to describe the average controllability in VN. (E) Brain map shows the regions with omnibus differences in modal controllability under the 2-back load. (F) Radar map exhibits the modal controllability values in detected regions for each group. Similarly, the mean values of modal controllability of all detected nodes in the corresponding network are used if the network has several nodes. (G) Pie chart shows the proportion of detected nodes in each large-scale network. *P_FDR_ < 0.05; **P_FDR_ < 0.01; ***P_FDR_ < 0.001.

For average controllability (Fig. 2A to D and Table S1), under the 0-back load, SZ exhibited lower average controllability in the memory retrieval network (MRN; one node; corresponding region: right posterior cingulate cortex) than MDD and HCs. Under the 2-back load, SZ and MDD had lower average controllability in 2 nodes of the visual network (VN; 3 nodes; right cuneus, left lingual gyrus, and right middle occipital gyrus) than BD and HCs; SZ had lower average controllability in the FPN (one node; right inferior temporal gyrus) than other 3 groups.

For modal controllability, no significant difference was found under the 0-back load. Under the 2-back load (Fig. 2E to G and Table S1), SZ showed lower modal controllability in multiple network nodes than HCs, including FPN (12 nodes; left inferior frontal gyrus, bilateral inferior parietal lobule, and etc.), salience network (SN; 3 nodes; bilateral insula and left median cingulate and paracingulate gyri), DMN (2 nodes; right angular gyrus and left inferior frontal gyrus), sensorimotor network (SMN; 2 nodes; left postcentral gyrus), auditory network (AN; 1 node; left postcentral gyrus), VN (1 node; left inferior occipital gyrus), cingulo-opercular network (CON; 1 node; right medial frontal gyrus), cerebellum (1 node; right cerebellum posterior lobe), and dorsal attention network (DAN; 1 node; left middle frontal gyrus). Notably, SZ, BD, and MDD all exhibited lower modal controllability in partial nodes of FPN than HCs. SZ and BD shared lower modal controllability in one node of SMN than HCs; SZ and MDD shared lower modal controllability in one node of SN than HCs.

### Clinical and cognitive correlation of abnormal average and modal controllability

**Fig. 3.**
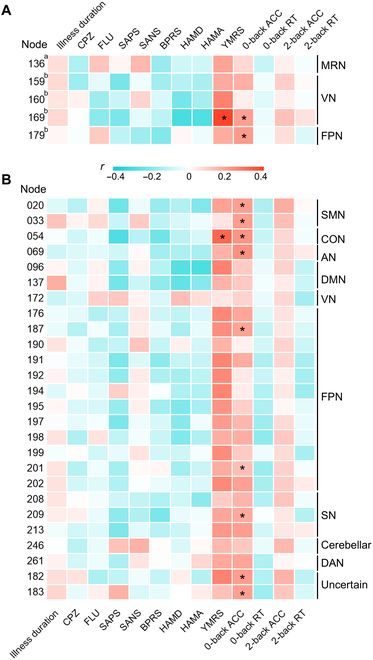
Clinical and cognitive correlation of abnormal average controllability and modal controllability. (A) Heatmap depicts the correlation between regions with omnibus differences in average controllability and clinical and cognitive characteristics under 0-back and 2-back loads. (B) Heatmap depicts the correlation between regions with omnibus differences in modal controllability and clinical and cognitive characteristics under the 2-back load. The index number of the node and corresponding network are based on the Power atlas. ACC, accuracy; RT, response time. a indicates under the 0-back load; b indicates under the 2-back load. *P_FDR_ < 0.05.

Under the 2-back load, average controllability of the VN node (node 169; right middle occipital gyrus) was positively correlated with YMRS scores [*r* = 0.39, false-discovery-rate-corrected *P* (*P*_FDR_) = 0.010] and 0-back target accuracy (*r* = 0.20, *P*_FDR_ = 0.036). The FPN node (node 179; right inferior temporal gyrus) was also positively correlated with the 0-back target accuracy (*r* = 0.21, *P*_FDR_ = 0.036), as shown in Fig. 3A and Table S2.

We observed positive correlations between the modal controllability of the CON node (node 54; right medial frontal gyrus) and YMRS scores (*r* = 0.32, *P*_FDR_ = 0.036) and 0-back target accuracy (*r* = 0.23, *P*_FDR_ = 0.027). The 0-back target accuracy was also positively associated with the modal controllability of 2 nodes in SMN [node 20: *r* = 0.20, *P*_FDR_ = 0.036; node 33: *r* = 0.20, *P*_FDR_ = 0.036 (left postcentral gyrus)], the AN node (node 69: *r* = 0.23, *P*_FDR_ = 0.022; left postcentral gyrus), 2 nodes in FPN [node 187: *r* = 0.19, *P*_FDR_ = 0.047 (left inferior frontal gyrus); node 202: *r* = 0.20, *P*_FDR_ = 0.036 (left superior frontal gyrus)], and the SN node (node 213: *r* = 0.20, *P*_FDR_ = 0.036; left median cingulate and paracingulate gyri), as shown in Fig. 3B and Table S3. We found no significant correlations between illness duration and chlorpromazine (CPZ)/fluoxetine (FLU) dose and abnormal controllability.

### Spatial correlation analysis

**Fig. 4.**
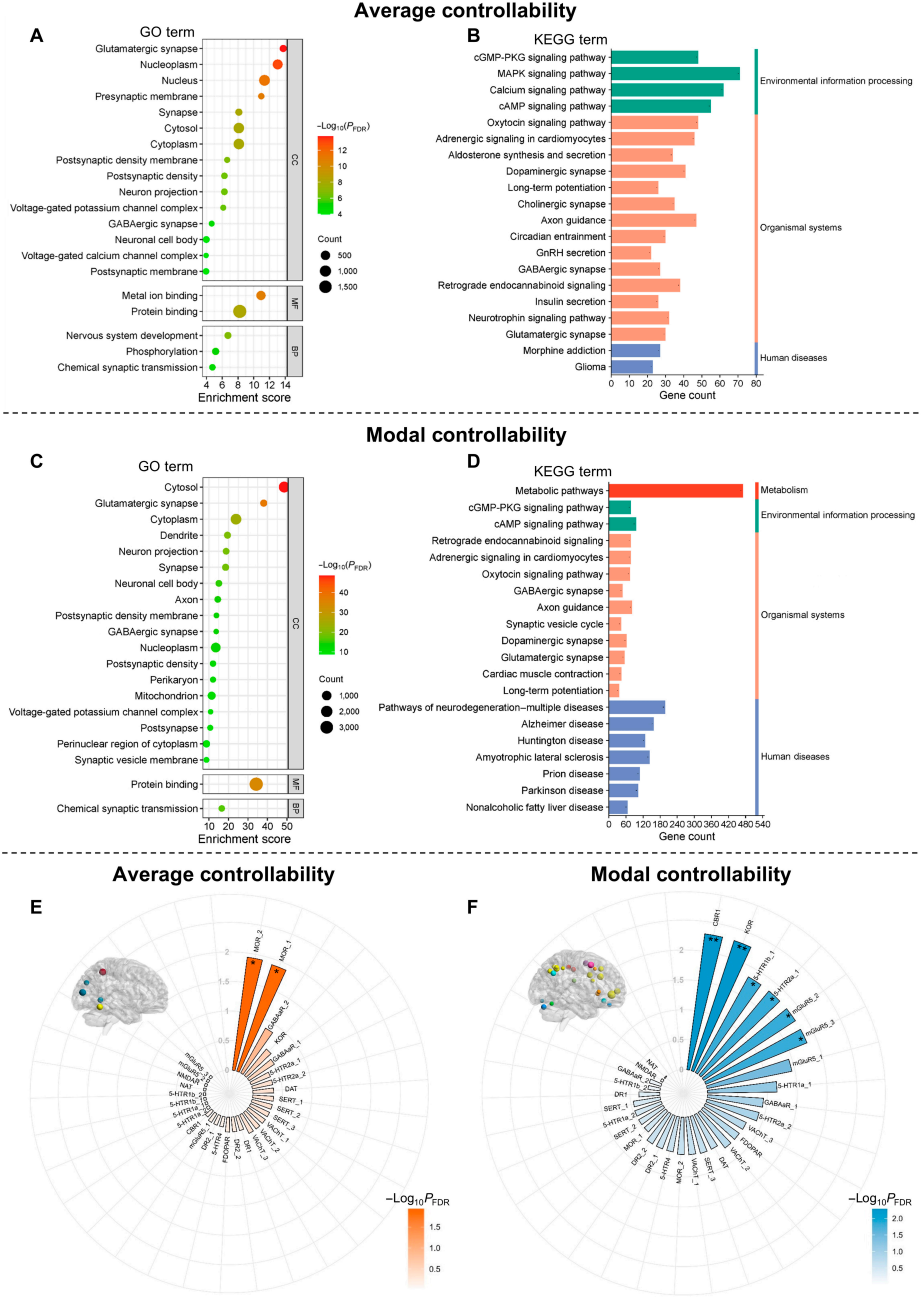
Spatial correlation between transcriptome and neurotransmitter and abnormal average controllability and modal controllability. (A and C) The top 20 significantly enriched GO terms associated with abnormal average and modal controllability. (B and D) The top 20 significantly enriched KEGG terms associated with abnormal average and modal controllability. cGMP, guanosine 3′,5′-monophosphate; PKG, cGMP-dependent protein kinase; MAPK, mitogen-activated protein kinase; cAMP, adenosine 3′,5′-monophosphate; GnRH, gonadotropin-releasing hormone. (E and F) Neurotransmitters associated with abnormal average (E) and modal controllability (F). BP, biological process; CC, cellular component; MF, molecular function; SERT, serotonin transporter; D, dopamine; DAT, dopamine transporter: FDOPA. fluorodopa; NAT, noradrenaline transporter; NMDA, *N*-methyl-d-aspartic acid; SERT, serotonin transporter; VAChT, vesicular acetylcholine transporter. *P_FDR_ < 0.05 and **P_FDR_ < 0.01.

The genetic annotation analyses identified 3,245 and 6,202 statistically differentially expressed genes associated with detected regions of omnibus abnormal average and modal controllability respectively (*P*_FDR_ < 0.05). Enrichment results of Gene Ontology (GO) terms for abnormal average and modal controllability were similar. These genes were mainly enriched in GO terms of synapse-related cellular components, such as “glutamatergic synapse”, “synapse”, and “γ-aminobutyric acid-ergic (GABAergic) synapse” (all *P*_FDR_ < 0.05; Fig. 4A and C). In the enrichment results of the Kyoto Encyclopedia of Genes and Genomes (KEGG) pathway, we noted that differentially expressed genes for abnormal average and modal controllability were both enriched in organismal systems, such as glutamatergic synapse, “dopaminergic synapse”, and GABAergic synapse; in addition, the expressed genes for abnormal modal controllability were also enriched in the “metabolic pathways” (all *P*_FDR_ < 0.05; Fig. 4B and D).

We further investigated spatial relationships between neurotransmitter systems and omnibus abnormal average and modal controllability, respectively. The average controllability was negatively associated with the availability of mu opioid receptors (MOR_1: *r* = −0.31, *P*_FDR_ = 0.013; MOR_2: *r* = −0.33, *P*_FDR_ = 0.012), as shown in Fig. 4E and Table S4. The abnormal modal controllability overlapped with regions that had higher availability of 5-hydroxytryptamine (5-HT; 5HT1b_1: *r* = 0.28, *P*_FDR_ = 0.015; 5HT1a_2: *r* = 0.27, *P*_FDR_ = 0.015), cannabinoid type 1 (CB1: *r* = 0.33, *P*_FDR_ = 0.005), metabotropic glutamate type 5 (mGluR5_1: *r* = 0.24, *P*_FDR_ = 0.035; mGluR5_2: *r* = 0.27, *P*_FDR_ = 0.015; mGluR5_3: *r* = 0.28, *P*_FDR_ = 0.015), and kappa opioid receptor (KOR: *r* = 0.35, *P*_FDR_ = 0.005), as shown in Fig. 4F and Table S4.

In addition, we also performed spatial correlation analyses for average and modal controllability with illness-specific (difference found in only one patient group compared to HCs) and transdiagnostic (difference shared across 2 or all 3 patient groups compared to HCs) features and under each task load. For genetic results, no significant correlation was found for SZ-specific average controllability under the 0-back load. Under the 2-back load (Figs. S2 to S7), differentially expressed genes associated with both SZ-specific and transdiagnostic average and modal controllability were all mainly consistent with our main results. For transmitter results (Tables S5 and S6), significant correlations were found only for SZ-specific abnormal modal controllability under the 2-back load, with the availability of the serotonin transporter, fluorodopa receptor, and glutamate receptor (Table S5).

## Discussion

The present study unveiled brain controllability patterns during WM tasks among MPDs, with a potential link to gene and neurotransmitter profiles. SZ showed reduced controllability in all detected regions (mainly encompassing FPN, DMN, SN, MRN, and sensory networks) compared with HCs, suggesting that the generalized reduction in the capacity to control transition dynamics during the WM task is most pronounced in SZ among MPDs. We highlight 3 more specific findings. (a) Under the 2-back load, SZ, BD, and MDD all exhibited lower modal controllability in partial nodes of FPN than HCs. SZ and MDD shared lower modal controllability of the SN node relative to HCs. (b) SZ and MDD shared lower average controllability of VN nodes than BD and HCs. SZ had lower modal controllability of SMN, AN, and VN nodes than HCs. Correlation analyses revealed that lower average and modal controllability of FPN, SN, VN, SMN, and AN nodes was associated with poor WM task performances. (c) Differentially expressed genes associated with abnormal controllability may be involved in distributed synaptic functions within the glutamatergic and GABAergic systems. There were significant associations between abnormal controllability and specific neurotransmitter systems including glutamate, opioid, 5-HT, and cannabinoid receptors.

We noted that under the 0-back load, only SZ showed lower average controllability in the MRN (right posterior cingulate gyrus) than HCs. The MRN, crucial for autobiographical (personally experienced) episodic memory [31] and sharing functional similarities with the DMN (processing self-referential information), is typically suppressed during external goal-directed tasks such as WM [32]. Our finding suggests an impaired ability of MRN to transition from a resting/self-referential state to a general task-processing state in SZ. With the increase in task load, patient groups, especially SZ, had altered controllability in distributed brain areas. Increased complexity/load of WM task likely demands more manipulation of cognitive resources, as evidenced by extensive functional recruitment [33]. Most areas recognized for their role in WM exhibited load-dependent activity and connectivity when performing WM tasks in SZ [34]; our results are in concordance with this literature.

We speculate that widespread controllability deficits under the 2-back (contrast to the 0-back) may stem from 2 mechanisms linked to increased cognitive demand. First, high cognitive load under the 2-back may exceed metabolic capacity, particularly for adenosine 5′-triphosphate (ATP)-dependent synaptic plasticity critical to cognitive control [35,36]. Brain energy consumption surges during intense tasks, and if ATP production cannot match demands for synaptic maintenance/modification, neural signaling fails, compromising controllability. Second, we observed prominent controllability deficits in FPN under the 2-back, with SZ showing the lowest average controllability of FPN across the 4 groups and lower modal controllability in multiple FPN nodes than HCs. FPN plays an important role in the top-down modulation of cortical dynamic activity during preparatory attention and orientation within WM [37]. Different cognitive control processes are hierarchically organized and require coordinated resource allocation in the prefrontal cortex and related networks [38]. Therefore, inefficient FPN-driven coordination under the 2-back likely underlies controllability deficits in SZ. This is consistent with prior reports of impaired functional activation and connectivity in FPN during WM tasks in SZ [39,40]. Our previous work [15] reported that SZ had increased temporal variability of degree centrality in the inferior parietal lobe (a key FPN node in our results) under the 2-back load. We hypothesize that the excessive neural dynamics observed in SZ may lead to instability and an inability to effectively organize and modulate brain activity to achieve desirable brain states.

Among MPDs, SZ had the illness-specific low average controllability of FPN, and the low modal controllability of FPN appears to be a transdiagnostic trait. Average controllability focuses on the ability of driving brain states to expected easy-to-reach states, and modal controllability reflects the ability to transition toward difficult-to-reach states. We speculated that in BD and MDD, FPN only showed abnormal functional dynamics during complex cognitive processes. In a previous transdiagnostic study, MPDs showed reduced functional connectivity of FPN compared with HCs, and patients with psychosis had reduced connectivity of FPN compared with patients without psychosis during rest [41]. This finding implicates a shared functional dysconnectivity of FPN across MPDs, with a more pronounced dysconnectivity in psychosis. Our results indicate that brain controllability may serve as a more sensitive metric for detecting both transdiagnostic and SZ-specific functional features of FPN during WM tasks among MPDs.

We noted that SZ had lower modal controllability of DMN than HCs; SZ and MDD shared lower modal controllability of SN than HCs under the 2-back load. Lower controllability of FPN and SN nodes were associated with poor WM task performances. A previous resting-state fMRI study also suggests the correlation between abnormal controllability of SN and FPN and psychosis symptoms in SZ [20]. The classical triple network model of cognitive control s4ystem emphasizes the key role of coordination of the DMN, SN, and FPN in the execution of WM [42]. It is proposed that SN facilitates switching brain networks, resulting in the engagement of FPN to manage oriented attention and disengagement of the DMN to be involved in oriented self-referential mental processes during WM tasks [43]. Our results indicated that this cognitive triple network model less readily shifts to expected difficult-to-reach states, which is consistent with previous work on functional dysconnectivity of triple network shared in MDD and SZ [44]. The disrupted dynamics of the triple network may contribute to negative symptoms in SZ, as well as psychomotor retardation in MDD.

Differences of controllability in sensory networks (SMN, AN, and VN) had also been observed among groups under the 2-back load. SZ and MDD showed lower average controllability of VN than BD and HCs. SZ had lower modal controllability of SMN, AN, and VN than HCs, and other patient groups did not differ from HCs, indicating that activity of the sensory networks may less readily drive brain state transition to desirable states necessary for WM tasks. This might be related to the abnormal activation and connectivity of sensory systems under WM in SZ [34,45]. The average controllability of SMN and VN has been indicated to be linked to psychotic and depressive symptoms [20,46]. Deficits in sensory networks may impair the effective processing and integration of external sensory information with higher-order systems [47]. Our results highlight that SZ exhibited a notable transition disturbance of the sensory cortex, which is crucial for cognitive tasks.

We integrated the gene expression, transcription, and neurotransmitter analyses to interpret the molecular mechanisms underlying network controllability. Neurotransmission is partially driven by gene expression profiles, and neurotransmitter imbalances can disrupt neural signaling and activity, thereby impairing cognitive function [48,49]. Our results demonstrated that the relevant gene expression primarily involved glutamatergic, GABAergic, and dopaminergic synaptic systems. Neurotransmitter correlation analysis further revealed that glutamate and 5-HT were associated with abnormal controllability.

Classical neurotransmitter hypotheses for the pathophysiology of MPDs posit dysregulated neurotransmission in dopamine and 5-HT pathways, along with the excitatory–inhibitory imbalance via glutamate and γ-aminobutyric acid (GABA) [50–52]. Arnsten et al. [53] demonstrated that the prefrontal cortex microcircuitry for WM relies on excitatory–inhibitory balance of glutamatergic and GABAergic neurons, dynamically regulated by neuromodulators such as dopamine. Glutamatergic levels increased with WM load in HC but not in SZ or BD, reflecting a dysfunction of glutamatergic systems in SZ and BD under high cognitive demands [54]. This finding aligns with our observation of neurotransmitter system associations specifically with 2-back (versus 0-back) controllability deficits. Notably, altered GABAergic signaling may reflect adaptive adjustments to network hyperexcitability. Ragland et al. [55] found negative relationship between GABA levels in dorsolateral prefrontal cortex (DLPFC) and WM performance in SZ, suggesting that regional GABAergic changes might mitigate, rather than drive, controllability impairments. Our findings suggested that disruptions in these classical neurotransmitter pathways may underlie neural dysfunction of WM deficits in MPDs.

The above findings implicate disruptions in classical neurotransmitter pathways as a basis for neural dysfunction underlying WM deficits in MPDs. Future multimodal studies [e.g., PET and magnetic resonance spectroscopy (MRS)] are crucial to elucidate the specific mechanisms. A multimodal fMRI-MRS study in unmedicated SZ showed correlations between DLPFC glutamate levels and WM-induced neural activation [56], which aligns with our observation of reduced modal controllability in DLPFC nodes within FPN in SZ. Integrating molecular topology (e.g., transcriptomic gradients) could facilitate mechanistic mapping of region-specific receptor densities to network-specific controllability deficits [57].

Several limitations need to be considered. First, the sample size of this study is relatively small, so future studies with larger cohorts are needed to verify our findings. Three patient groups showed significant differences in the age and education years. Despite incorporating these variables as covariates in the controllability analyses, they may still introduce confounding effects. Second, the BD group comprised patients in various states—depressive, manic/hypomanic, mixed, and euthymic states. While this inclusion provides a more comprehensive view of BD, it introduces a degree of heterogeneity that may obscure state-specific brain controllability patterns associated with each clinical presentation. Third, as this study calculated controllability using only fMRI data, future investigations of structural controllability (e.g., via diffusion-tensor-imaging-derived networks) would provide complementary insights into the anatomical foundations of brain state transitions. A previous study revealed that structural controllability was positively correlated with functional participation coefficient and played a mediating role in brain anatomical structure to support functional dynamics [58]. Fourth, future studies should use WM tasks with varying difficulty levels and dynamic network modeling to characterize load-dependent controllability patterns and clarify whether 2-back differences stem from adaptive failure or preexisting deficits. Moreover, this study lacked an independent validation sample, so our results need to be further validated by large and well-balanced populations.

## Conclusion

Aberrant brain network controllability during WM in MPDs affects the triple network (FPN, DMN, and SN) and sensory systems (SMN, AN, and VN) in SZ and other MPDs, and has a synaptic and neurotransmitter basis pertaining to glutamate, dopamine, GABA, and 5-HT, all of which play key roles in the clinical expression of MPDs. These findings indicate that a core dysfunction in the capacity to transition between network states may explain dysfunctional working memory in major psychiatric disorders.

## Materials and Methods

### Participants

This study adopted a cross-sectional design and recruited 110 patients with SZ, 70 patients with BD, and 55 patients with MDD from the Second Xiangya Hospital, Central South University and 82 HCs from the community from 2009 to 2017. All the patients were diagnosed by board-certified psychiatrists using the Diagnostic and Statistical Manual of Mental Disorders, Fourth Edition (DSM-IV) criteria for SZ, BD, and MDD. Patients aged 18 to 50 years old with at least 9 years of education were included. Exclusion criteria included neurological disorders, major physical illness, history of substance dependence, history of receiving electroconvulsive therapy, or any contraindications to MRI. The other criteria for states of BD are described in File S2. HCs were recruited from the local community through advertisement. The inclusion and exclusion criteria for HCs were the same as those for patients except that the HCs and their first-degree relatives did not have personal histories of any psychiatric disorders.

### Clinical assessments

We adopted the BPRS, Scale for the Assessment of Positive Symptoms (SAPS), and Scale for the Assessment of Negative Symptoms (SANS) to assess the severity of psychotic symptoms. The YMRS, HAMD, and HAMA were used to evaluate the severity of manic, depressive, and anxiety symptoms.

### MRI data acquisition and preprocessing

Imaging scans were performed on a Philips 3.0T scanner with an 8-channel head coil using a gradient-recalled echo-planar imaging pulse sequence. Data preprocessing was performed using the DPABI toolbox (http://www.rfmri.org/) [59]. Preprocessing included discarded 2 first images, slice timing correction, head motion realignment, spatial normalization to Montreal Neurologic Institute space, and smoothing. The imaging parameters and preprocessing details are presented in File S3.

### WM task paradigm

We applied the *n*-back task as the WM paradigm, which included 0-back and 2-back loads in this study. In the 0-back load, participants pressed a button once when they saw the letter “x”; in the 2-back load, participants pressed a button once when the letter presented was the same as 2 letters prior. A detailed description of this paradigm is given in File S4 and Fig. S1.

### Calculation of controllability metrics

We first constructed a functional connection matrix before calculating controllability metrics. Each block contains 20 volumes, and, thus, the 0-back and 2-back loads consist of 80 volumes, respectively. For each participant, we separately concatenated the 80 volumes obtained under the 4 blocks of the 0-back load and the 4 blocks of the 2-back load. We extracted the mean time series from each of the 264 nodes using 6-mm spheres defined by the Power atlas [60] and generated a 264 × 264 symmetric matrix for each participant by computing the Pearson correlation coefficients between the time series for each pair of nodes. The resultant matrix was converted to normally distributed scores by using Fisher’s *z* transformation.

Then, 2 commonly used metrics of network controllability, average controllability, and modal controllability [17,20] are calculated in the matrices constructed under 0-back and 2-back loads, respectively. Details of the calculation of these metrics are provided in File S5.

### Statistical analysis

The SPSS statistical software (version 22) was adopted to compare the demographic and clinical data and controllability metrics across groups. Differences in age, years of education, clinical data, and 0-back and 2-back task performances were analyzed using one-way analysis of variance (ANOVA), and sex differences were assessed using χ^2^ test (*P* <0.05). Controllability metrics of 264 nodes were compared using the analysis of covariance (ANCOVA) test with sex, age, years of education, and head motion as covariates. The 264 nodes were partitioned into various large-scale networks defined by the Power atlas [60]. We also performed correlation analysis to relate the detected regions with altered controllability with clinical and cognitive characteristics after controlling for age, gender, education, and head motion. The threshold of statistical significance was set at *P*_FDR_ < 0.05.

### Spatial correlation analysis

We further conducted imaging transcriptome analysis and neurotransmitter correlation analyses on the average and modal controllability maps of detected regions with omnibus differences across 4 groups.

#### Imaging transcriptome analysis

We used the Brain Annotation Toolbox (BAT) [61] to perform genetic annotation analysis on the observed regions with abnormal average and modal controllability [61]. The gene expression profiles could be extracted from the Allen Human Brain Atlas [25] via BAT based on the brain regions. The permutation analysis was conducted to identify differentially expressed genes within the specified regions compared to samples in the background [61], with permutation times of 5,000. The other parameters for genetic annotations were as follows: region of interest size = 6 mm and minimal sample size = 5. The statistical significance level was set as *P*_FDR_ < 0.05.

The derived differentially expressed genes were uploaded to the Database for Annotation, Visualization, and Integrated Discovery (https://david.ncifcrf.gov/). The GO database, specifically focusing on 3 domains, including the biological process, cellular component, and molecular function, and the KEGG database for *Homo sapiens* sets were adopted to achieve gene function and pathway enrichment analysis. The statistical significance level was set as *P*_FDR_ < 0.05.

#### Neurotransmitter correlation analysis

We adopted JuSpace to explore the neurochemical basis underlying controllability abnormalities. JuSpace is a practical tool for spatial correlation analyses of MRI with PET/SPECT-derived neurotransmitter receptor/transporter availability maps with different tracers (https://github.com/juryxy/JuSpace) [62], which has been previously used to explore neurochemical basis of neural correlates [63]. We calculated Pearson correlation coefficients between detected regions with abnormal average and modal controllability and various neurotransmitter maps including dopamine, serotonin, glutamate, GABA, acetylcholine, opioid, cannabinoid, noradrenaline, and fluorodopa, while adjusting for spatial autocorrelation and partial volume with the gray matter probability map [62]. The spatial permutation-based null maps with 5,000 permutations were used to compute exact *P* values. The statistical significance level was set as *P*_FDR_ < 0.05.

## Data Availability

The data that support the findings of this study are available from the corresponding authors upon reasonable request.

## Supplementary Materials

Supplementary 1 Files S1 to S5 Figs. S1 to S7 Tables S1 to S6 References [66–81]
