# Supplementary material for: Task-Related Controllability of Functional Connectome During a Working Memory Task in Schizophrenia, Bipolar Disorder, and Major Depressive Disorder
Source: Research (Wash D C). 2025 Aug 5;8:0792. doi: 10.34133/research.0792 (PMC12324819; doi:10.34133/research.0792)
Supplement: Supplementary 1 — Files S1 to S5 Figs. S1 to S7 Tables S1 to S6 References [66–81] [file research.0792.f1.docx]

### Supplementary Materials

**File S1.** **Controllability metrics: structure vs Function**

Controllability theory when first brought to brain imaging, was applied to structural brain networks, where connections represent physical pathways for neuronal influence, as demonstrated in the foundational work by Gu et al. ^[17]^. These early studies largely focused on understanding structure-constrained brain-state reconfigurations. While biophysically grounded on the structural pathways, this approach is limited by the assumption that “all effective relations between regions are time-invariant and encapsulated in the underlying white matter network architecture” ^[67]^. Tu and colleagues raised objections, partly on the grounds that structure-based control estimates cannot be mapped on to functional topology ^[68]^. While not directly invoking brain’s structure, Leitold highlighted that physical topologies do not relate to dynamical systems they purport to model, unless the edges have demonstrable functional relationship ^[69]^. Both in health and disease, several studies have demonstrate the lack of one-to-one correspondence between structural and functional pathways in the brain (i.e., “function cannot be directly estimated from structure” ^[70]^). In fact, Cai, Menon and colleagues have argued that control hubs identified from structure do not have the same functional control profile ^[71]^. Other constraints in making inferences from structural controllability are discussed elsewhere ^[72]^.

The successful application of network control theory hinges on the interaction matrix that describes how a system evolves from the current to the future state. Estimating the state of connectivity elicited by a given brain state from functional time series provides a more direct interpretation for the state-shifts that are modelled. When we use a functional connectivity for the interaction matrix in the control model, higher link weight is interpreted in the same way as structural matrix – i.e. signal progression along the links occur with stronger edge weight or diffusion. Nevertheless, as argued by Bassett and colleagues, functional edges do not have physical, but informational meaning ^[73]^. Throughout our manuscript, we have adhered to this non-physical interpretation.

We also wish to highlight a number of studies that successfully demonstrate both construct validity and reliability of fMRI-based controllability measures. For example, studies on schizophrenia ^[45]^ as well as siblings in our previous work ^[74]^, obsessive-compulsive disorder ^[75]^, dementia ^[76]^, glioma ^[77]^, MDD ^[78]^, and psychedelic states ^[79]^, and in healthy subjects ^[80]^. Our approach aligns with this paradigm, treating functional networks as models of dynamic inter-regional interactions, rather than mere similarity measures, that govern how brain states evolve in response to task demands.

**File S2. Supplementary criteria for states of BD**

In BD, consistent with our prior study ^[81]^, patients whose HAMD ≥17 and YMRS score <12 were considered depressive, patients whose HAMD <17 and YMRS score ≥12 were considered manic/hypomanic, patients whose HAMD ≥17 and YMRS score ≥12 were considered mixed, and patients whose HAMD score <17 and YMRS score <12 were considered euthymic.

**File S3. Detailed MRI data acquisition and preprocessing**

Imaging scans were performed on a Philips 3.0T scanner with an 8-channel head coil using gradient-recalled echo-planar imaging (EPI) pulse sequence. The parameters are as follows: volumes = 250, matrix size = 64 × 64, slices number = 36, slices thickness = 4mm, flip angle = 90°, field of view (FOV) = 240 × 240mm^2^, repetition time (TR) = 2000ms, and echo time (TE) = 30ms.

Data preprocessing was conducted on the DPABI toolbox (DPABI, http://www.rfmri.org/)^[58]^. We discarded the first 2 volumes and the remaining 248 volumes were obtained for further preprocessing. The following preprocessing procedures included: slice timing correction, head motion realignment, spatial normalization to the Montreal Neurological Institute (MNI) space and smoothing (full width at half maximum [FWHM]=8mm). Nuisance covariates, including 12 head motion parameters, white matter signals, and ventricular signals, were regressed out. The global signal was retained due to potential disease-related variations. To reduce low-frequency drift and high-frequency physiological noise, a band-pass filter (0.08-0.15 Hz) was applied to the fMRI data. Displaced volumes (framewise displacement [FD] > 0.5 mm) were interpolated by nearest-neighbor interpolation. Participants with head motion more than 2.5mm translation or 2.5° rotation in any direction and fMRI data failed to normalize to MNI space were excluded. We included 105 SZ, 67 BD, 51 MDD, and 80 HCs in the final analysis. No significant differences were observed in the FD among groups (mean ± standard deviation: SZ=0.25±0.20, BD=0.22±0.11, MDD=0.25±0.17, HCs=0.20±0.08, *F*_(3,299)_=2.32, *p*=0.075).

**File S4. Detailed information of the working memory paradigm**

The adopted WM paradigm comprised “0-back” and “2-back” loads in this study. Four 0-back blocks, four 2-back blocks, and resting periods alternated throughout the whole task paradigm. In the “0-back”, participants pressed a button once when they saw the letter “x”; in the “2-back”, participants pressed a button once when the letter presented was the same as two letters prior. There was a stimulus interval of 1500ms between each 500ms letter display, and each block was composed of 20 stimuli containing 7 targets following an instruction shown for 2s (**Fig S1**). Participants were ordered to fixate on a cross in the center of a screen for 20s during resting periods.

**File S5. Controllability metrics**

Average controllability measures the ability of the brain area to drive the brain to easy-to-reach states while considering the average input energy cost. Brain areas with higher average controllability can smoothly facilitate the transition of functional states with lower input energy, suggesting they can more readily be transitioned into easy-to-reach states.

Modal controllability quantifies the ease with which a single control node can drive the brain into difficult-to-reach states. Regions with higher modal controllability can more easily guide the dynamics of a brain network toward difficult -to-reach states, incurring high energy costs for executing complex, goal-specific operations.

$$\begin{aligned} x\left( t+1 \right)=A\cdot x\left( t \right)+B_{K}\cdot u_{K}\left( t \right)\#\left( AUTONUM \backslash* Arabic \right) \end{aligned}$$

where $x\in R^{N}$ (*N*=246) describe the brain state at a given moment representing the magnitude of BOLD activity, *t* $\in$ $R$ [range: $(0, \infty)$] is the time interval of a state change in the discrete control systems. We take τ from 0 to infinity because prior work ^[82]^ has indicated that any state transitions can be achieved within a specific time from 0 to infinity and the infinity setup provides convenience in the calculation. $A\in R^{NxN}$ is the weighted and symmetric adjacency matrix. *A* is calculated by the following formula,

$$\begin{aligned} A= \frac{FC}{1+\lambda_{max}}-I\#\left( AUTONUM \backslash* Arabic \right) \end{aligned}$$

where $\lambda_{max}$ is the largest eigenvalue of the FC matrix, and *I* is the identity matrix. And then the estimated *A* can be used in equation $(1)$. We followed the setup in related work ^[83, 84]^.

The control input matrix $B_{K}\in R^{N\times m}$ represents a set of control points *K* in the brain where

$$K=\left\{ k_{1},\ldots,k_{m} \right\}, B_{K}=[e_{ki}\ldots e_{km}]$$

in which $e_{i}$ is the $i^{th}$ canonical vector of dimension *N*. In our case, 264 ROIs were set as control points. We set one ROI as a control point at a time, thus matrix *B* is a one-dimensional vector, e.g., $B_{K}$= (1 0 0 0…) when the first brain region is the control point. The $u_{K}\in R^{m}$ is the energy applied to the control point to drive state transition, which can be calculated by the following equation ^[85]^:

$$\begin{aligned} u\left( t \right)=B^{T}e^{A^{T\left( t_{f}-t \right)}}W^{-1}\left[ t_{0},t_{f} \right]d \#\left( AUTONUM \backslash* Arabic \right) \end{aligned}$$

We note that we don’t need to calculate the value of $u\left( t \right)$. $u\left( t \right)$ is proportional to the inverse of the controllability Gramian ($W_{K}^{-1}$). The purpose of quantifying the control properties of brain regions can be achieved by directly estimating *W* ^[82]^.

Brain network controllability reflects the possibility of driving the current state to the desired target state with external control energy input. A network is considered controllable if it can be driven from an initial state to other target states within a finite time. The controllability of a brain network with *K* control points equals the controllability Gramian matrix ($W_{K}$), in which

$$\begin{aligned} W_{K}= \sum_{\tau=0}^{\infty} A^{\tau}B_{K}B_{K}^{T}A^{\tau}\#\left( AUTONUM \backslash* Arabic \right) \end{aligned}$$

$A^{\tau}$ means the multiplication of A matrix (A$\times$A$\times$A... $\times$A) for *τ* times. The average input energy required for control points to drive the system is proportional to the trace of the inverse of $W_{K}$.

Modal controllability of control node *i* ($\emptyset_{i}$) is defined as:

$$\begin{aligned} \emptyset_{i}= \sum_{j=1}^{\infty} \left( 1-\lambda_{j}^{2}\left( A \right) \right)v_{ij}^{2}\#\left( AUTONUM \backslash* Arabic \right) \end{aligned}$$

where $\lambda_{j}$denotes the *j*^th^ eigenvalue of adjacency matrix *A*. $V=[v_{ij}]$ is the eigenvector matrix of *A*.


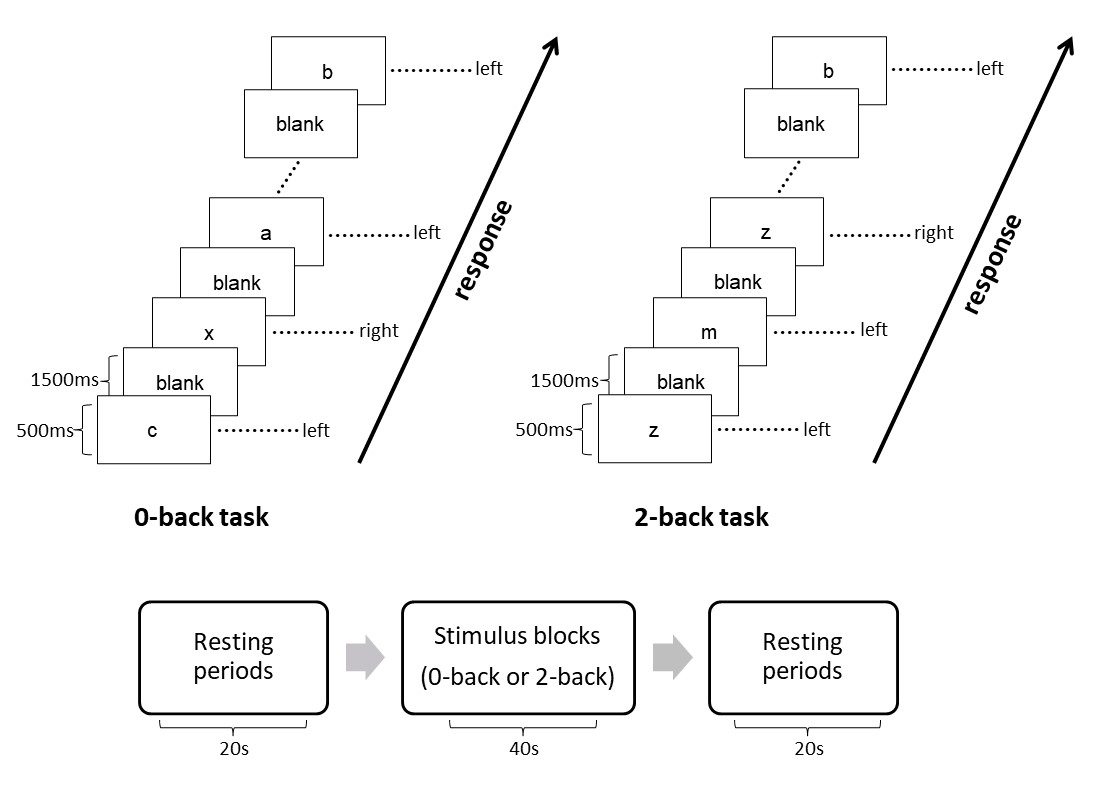
**Figure S1. The paradigm of n-back task**

**
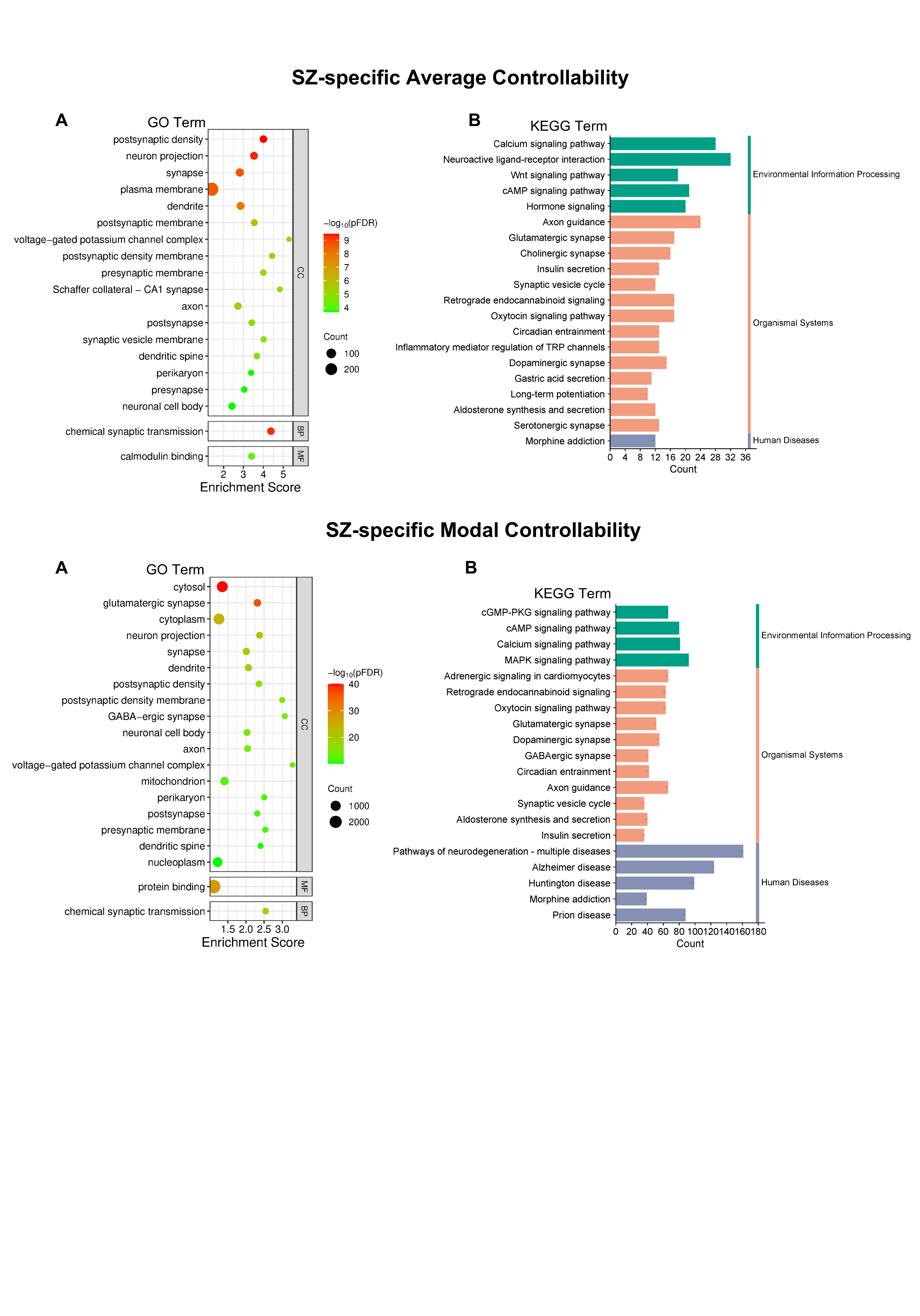
Fig S2. Spatial correlation between SZ-specific abnormal average controllability under the “2-back” load and transcriptome A) GO and B) KEGG.**

**
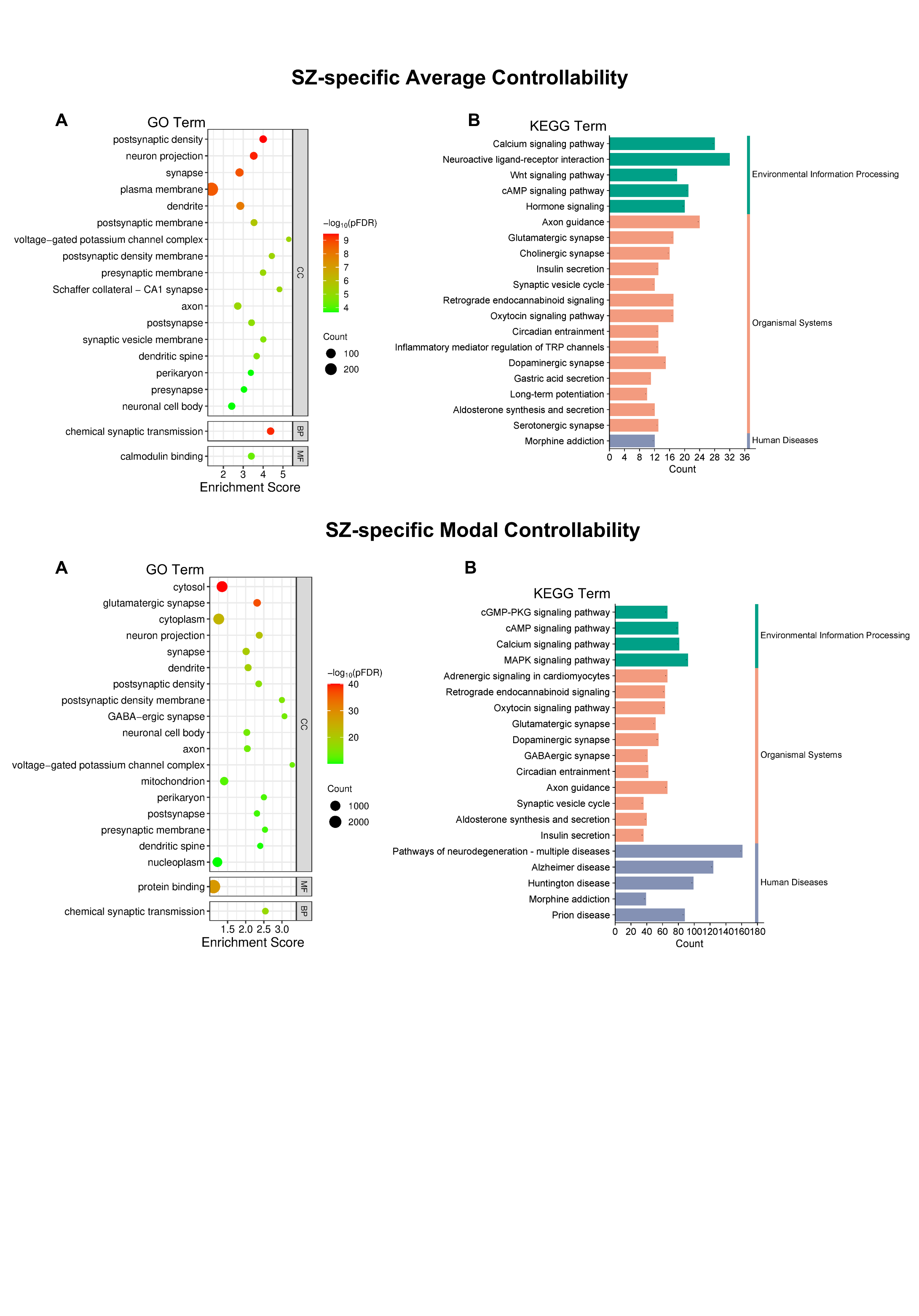
Fig S3. Spatial correlation between SZ-specific abnormal modal controllability under the “2-back” load and transcriptome A) GO and B)** **KEGG**.

**
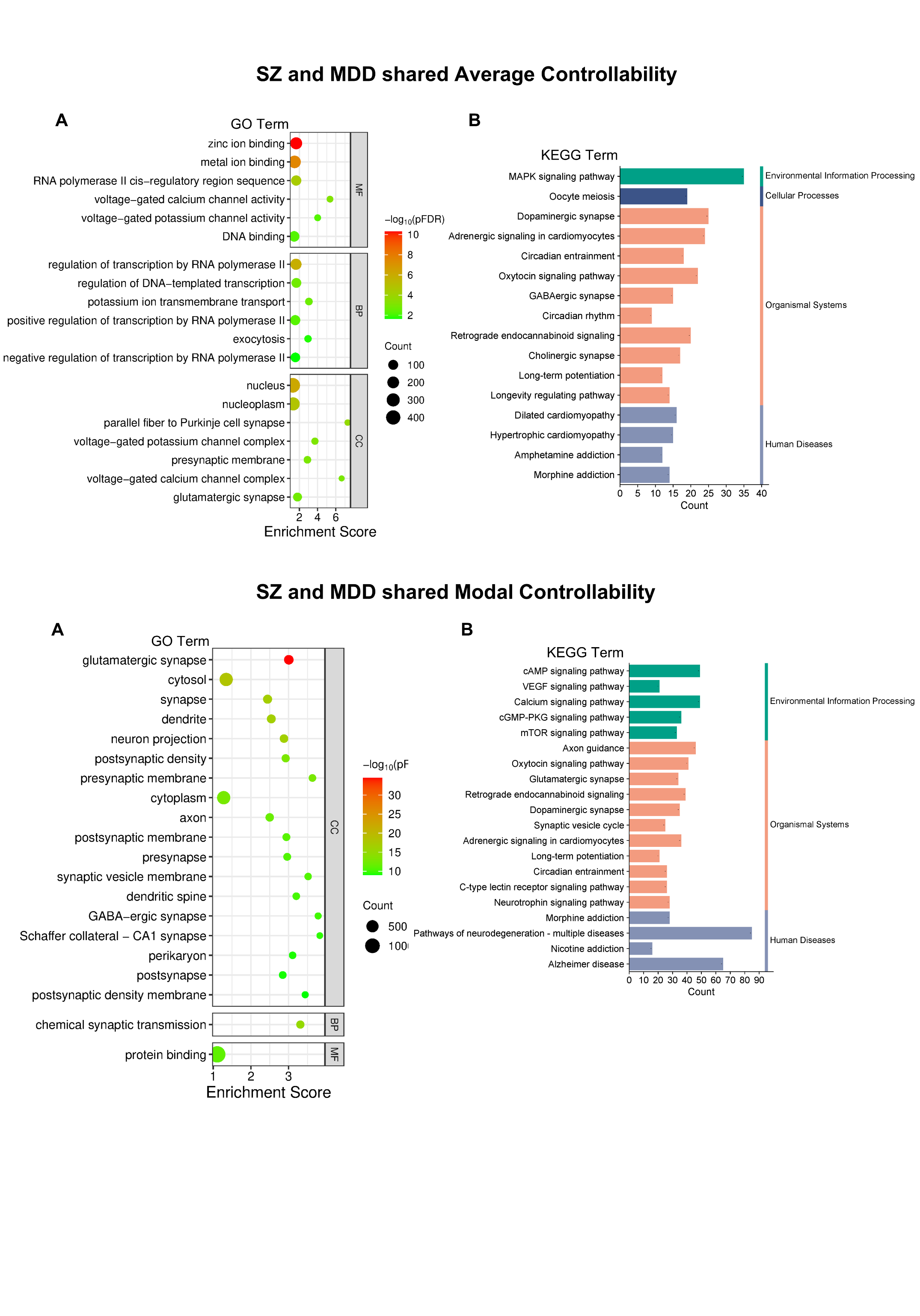
**

**Fig S4. Spatial correlation between SZ and MDD shared abnormal average controllability under the “2-back” load and transcriptome A) GO and B)** **KEGG**.

**
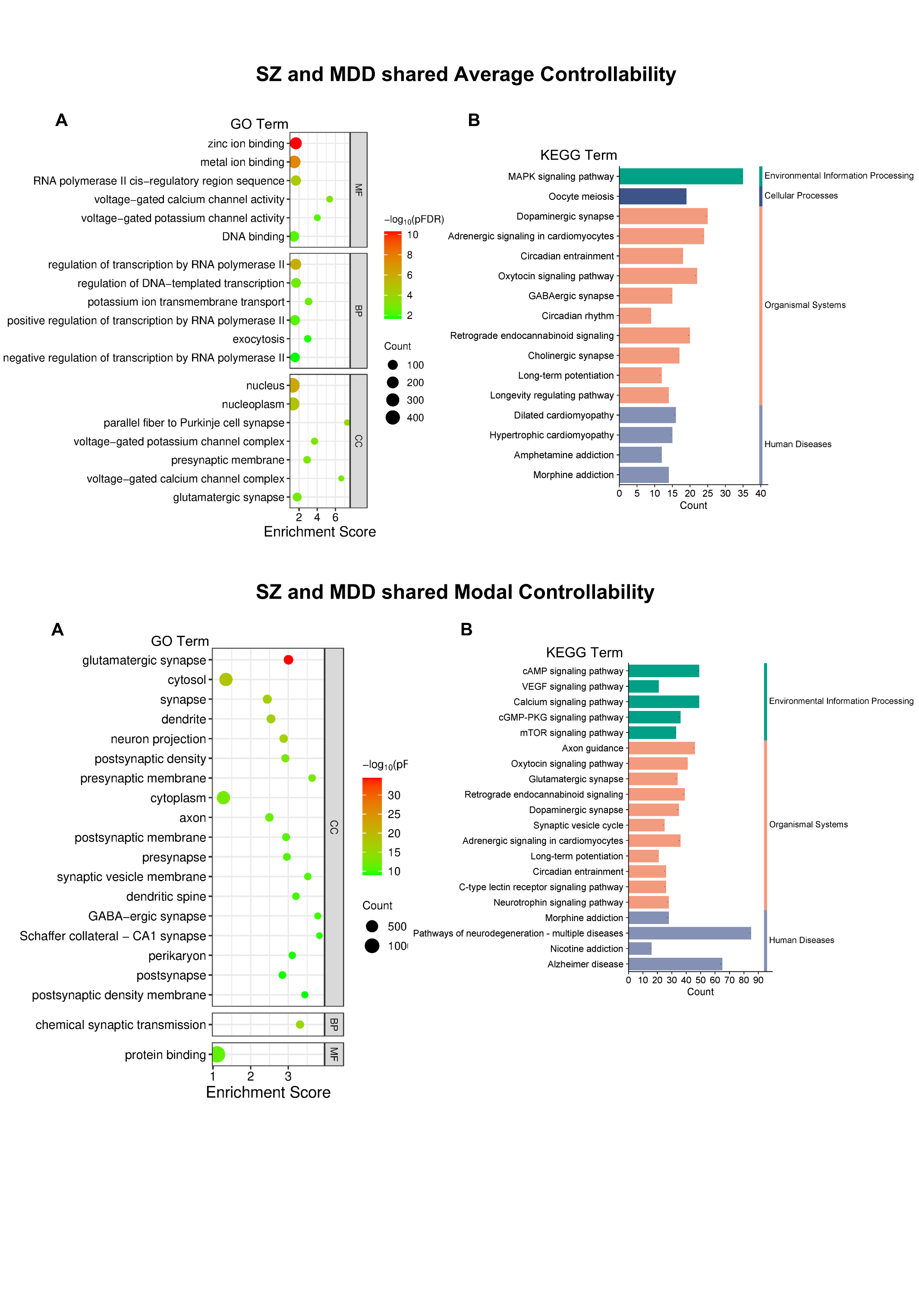
**

**Fig S5. Spatial correlation between SZ and MDD shared abnormal modal controllability under the “2-back” load and transcriptome A) GO and B)** **KEGG**.

**
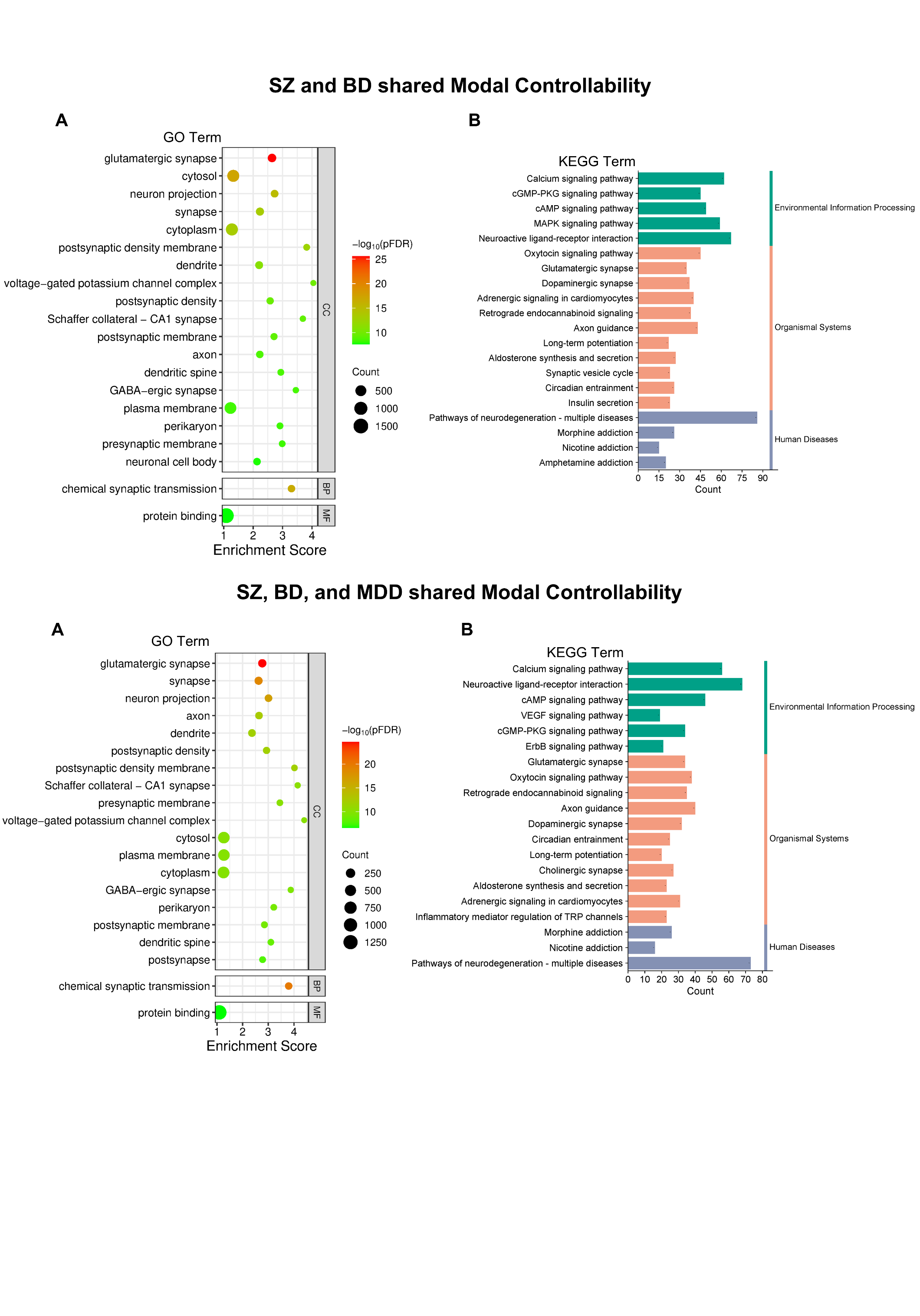
**

**Fig S6. Spatial correlation between SZ and BD shared abnormal modal controllability under the “2-back” load and transcriptome A) GO and B)** **KEGG**.

**
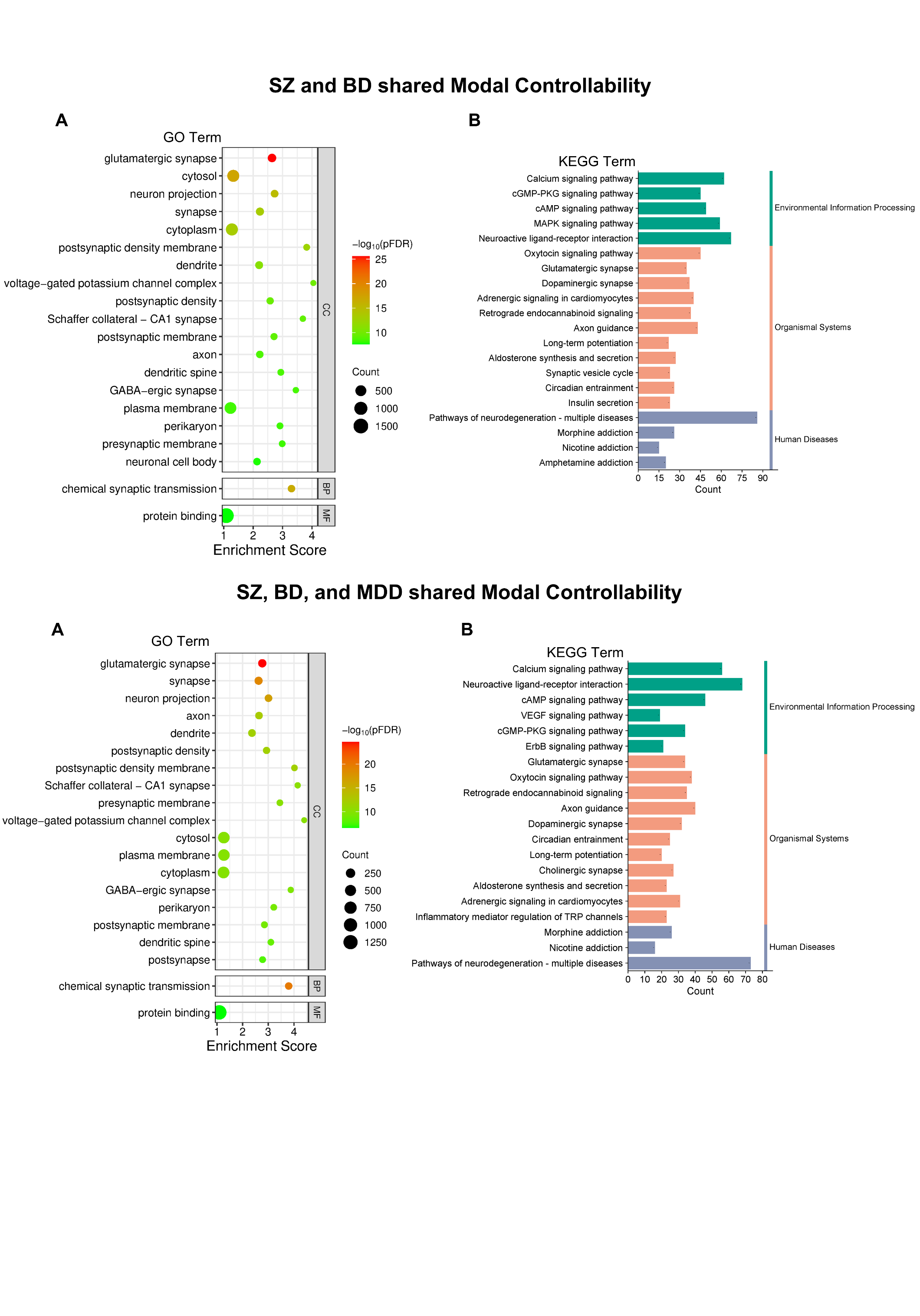
**

**Fig S7. Spatial correlation between SZ, BD, and MDD shared abnormal modal controllability under the “2-back” load and transcriptome A) GO and B)** **KEGG**.

**Table S1. Significant differences in average** **and modal controllability under** **“0-back” and “2-back” loads among all groups**

| **Node** | **Network** | **Brain Regions** | **MNI** | | | ***F*** | ***p*_FDR_** | **Post hoc analysis** | | | |  |
| --- | --- | --- | --- | --- | --- | --- | --- | --- | --- | --- | --- | --- |
|  |  |  | **X** | **Y** | **Z** |  |  | **SZ vs HCs** | **BD vs HCs** | **MDD vs HCs** | **SZ vs BD vs MDD** | |
| **Average controllability under “0-back” load** | | |  |  |  |  |  |  |  |  |  | |
| 136 | MRN | R Posterior Cingulate Cortex | 4 | -48 | 51 | 7.86 | 0.013 | SZ<HCs^**^ |  |  | SZ<MDD^***^ | |
| **Average controllability under “2-back” load** | | |  |  |  |  |  |  |  |  |  | |
| 159 | VN | R Cuneus | 15 | -77 | 31 | 6.14 | 0.030 | SZ<HCs^**^ |  |  | SZ<BD^**^ | |
| 160 | VN | L Lingual Gyrus | -16 | -52 | -1 | 6.51 | 0.025 | SZ<HCs^*^ |  | MDD<HCs^**^ | MDD<BD^**^ | |
|  |  |  |  |  |  |  |  |  |  |  |  | |
| 169 | VN | R Middle Occipital Gyrus | 37 | -84 | 13 | 7.73 | 0.015 | SZ<HCs^**^ |  | MDD<HCs^*^ | SZ<BD^**^, MDD<BD^*^ | |
|  |  |  |  |  |  |  |  |  |  |  |  | |
| 179 | FPN | R Inferior Temporal Gyrus | 58 | -53 | -14 | 7.11 | 0.017 | SZ<HCs^**^ |  |  | SZ<BD^*^, SZ<MDD^**^ | |
| **Modal controllability under “2-back” load** | | |  |  |  |  |  |  |  |  |  | |
| 20 | SMN | L Postcentral Gyrus | -54 | -23 | 43 | 5.41 | 0.020 | SZ<HCs^**^ | BD<HCs^*^ |  |  | |
| 33 | SMN | L Postcentral Gyrus | -45 | -32 | 47 | 5.51 | 0.020 | SZ<HCs^**^ |  |  |  | |
| 54 | CON | R Medial Frontal Gyrus | 7 | 8 | 51 | 7.23 | 0.008 | SZ<HCs^***^ |  |  | SZ<BD^*^ | |
| 69 | AN | L Postcentral Gyrus | -53 | -22 | 23 | 5.07 | 0.029 | SZ<HCs^**^ |  |  |  | |
| 96 | DMN | R Angular Gyrus | 52 | -59 | 36 | 6.08 | 0.013 | SZ<HCs^***^ |  |  |  | |
| 137 | DMN | L Inferior Frontal Gyrus | -46 | 31 | -13 | 5.67 | 0.019 | SZ<HCs^**^ |  |  | SZ<BD^*^ | |
| 172 | VN | L Inferior Occipital Gyrus | -33 | -79 | -13 | 4.63 | 0.040 | SZ<HCs^**^ |  |  |  | |
| 176 | FPN | L Inferior Frontal Gyrus | -47 | 11 | 23 | 4.65 | 0.040 | SZ<HCs^**^ |  |  |  | |
| 187 | FPN | L Inferior Frontal Gyrus | -41 | 6 | 33 | 6.42 | 0.012 | SZ<HCs^***^ |  | MDD<HCs^*^ |  | |
| 190 | FPN | R Inferior Parietal Lobule | 49 | -42 | 45 | 4.43 | 0.048 | SZ<HCs^**^ |  |  |  | |
| 191 | FPN | L Superior Parietal Lobule | -28 | -58 | 48 | 4.61 | 0.040 | SZ<HCs^**^ |  |  |  | |
| 192 | FPN | R Inferior Parietal Lobule | 44 | -53 | 47 | 4.97 | 0.031 | SZ<HCs^**^ |  |  |  | |
| 194 | FPN | R Inferior Parietal Lobule | 37 | -65 | 40 | 5.60 | 0.019 | SZ<HCs^**^ |  | MDD<HCs^**^ |  | |
| 195 | FPN | L Inferior Parietal Lobule | -42 | -55 | 45 | 5.82 | 0.017 | SZ<HCs^***^ |  |  |  | |
| 197 | FPN | L Middle Frontal Gyrus | -34 | 55 | 4 | 7.14 | 0.008 | SZ<HCs^***^ | BD<HCs^**^ | MDD<HCs^*^ |  | |
| 198 | FPN | L Middle Frontal Gyrus | -42 | 45 | -2 | 9.29 | 0.002 | SZ<HCs^***^ | BD<HCs^*^ |  |  | |
| 199 | FPN | R Inferior Parietal Lobule | 33 | -53 | 44 | 6.40 | 0.012 | SZ<HCs^***^ |  | MDD<HCs^**^ |  | |
| 201 | FPN | L Middle Frontal Gyrus | -42 | 25 | 30 | 7.40 | 0.008 | SZ<HCs^***^ | BD<HCs^*^ | MDD<HCs^**^ |  | |
| 202 | FPN | L Superior Frontal Gyrus | -3 | 26 | 44 | 6.13 | 0.013 | SZ<HCs^***^ |  |  |  | |
| 208 | SN | L Insula | -35 | 20 | 0 | 5.48 | 0.020 | SZ<HCs^**^ |  | MDD<HCs^*^ |  | |
| 209 | SN | R Insula | 36 | 22 | 3 | 4.86 | 0.034 | SZ<HCs^**^ |  |  |  | |
| 213 | SN | L Median cingulate and paracingulate gyri | -1 | 15 | 44 | 4.32 | 0.049 | SZ<HCs^**^ |  |  |  | |
| 246 | Cerebellum | R Cerebellum Posterior Lobe | 1 | -62 | -18 | 4.80 | 0.035 | SZ<HCs^**^ |  |  |  | |
| 261 | DAN | L Middle Frontal Gyrus | -32 | -1 | 54 | 6.50 | 0.012 | SZ<HCs^***^ |  |  |  | |
| 182 | Uncertain | L Middle Frontal Gyrus | -21 | 41 | -20 | 5.11 | 0.029 | SZ<HCs^**^ | BD<HCs^*^ |  |  | |
| 183 | Uncertain | L Cerebellum Posterior Lobe | -18 | -76 | -24 | 6.10 | 0.013 | SZ<HCs^***^ |  |  |  | |

Note: The index number of the node and corresponding brain regions and network are based on the Power Atlas.

Abbreviations: FDR, False Discovery Rate correction; Bon, Bonferroni correction; MRN, memory retrieval network; VN, visual network; FPN, frontoparietal network; SMN, sensory/somatomotor network; CON, cingulo-opercular network; AN, auditory network; DMN, default mode network; SN, salience network; DAN, dorsal attention network; MRN, memory retrieval network; VN, visual network; FPN, frontoparietal network; SMN, sensory/somatomotor network; CON, cingulo-opercular network; AN, auditory network; DMN, default mode network; SN, salience network; DAN, dorsal attention network;; L, left; R, right; SZ, Schizophrenia; BD, bipolar disorder; MDD, major depressive disorder; HCs, healthy controls. * *p*_Bonferroni_ < 0.05; ** *p*_Bonferroni_ < 0.01; *** *p*_Bonferroni_ < 0.001.

**Table S2. Clinical and cognitive correlation of nodes with abnormal average controllability under “0-back” and “2-back” loads**

| **ROI No.** | **Network** | **Illness duration** | | **CPZ** | | **FLU** | | **SAPS** | | **SANS** | | **BPRS** | | **HAMD** | | **HAMA** | | **YMRS** | | **0-back ACC** | | **0-back RT** | | **2-back ACC** | | **2-back RT** | |
| --- | --- | --- | --- | --- | --- | --- | --- | --- | --- | --- | --- | --- | --- | --- | --- | --- | --- | --- | --- | --- | --- | --- | --- | --- | --- | --- | --- |
|  |  | *r* | *p_FDR_* | *r* | *p_FDR_* | *r* | *p_FDR_* | *r* | *p_FDR_* | *r* | *p_FDR_* | *r* | *p_FDR_* | *r* | *p_FDR_* | *r* | *p_FDR_* | *r* | *p_FDR_* | *r* | *p_FDR_* | *r* | *p_FDR_* | *r* | *p_FDR_* | *r* | *p_FDR_* |
| **“0-back” load** | | | |  |  |  |  |  |  |  |  |  |  |  |  |  |  |  |  |  |  |  |  |  |  |  |  |
| 136 | MRN | 0.07 | 0.579 | -0.13 | 0.258 | 0.12 | 0.275 | 0.02 | 0.901 | 0.15 | 0.391 | -0.16 | 0.229 | 0.17 | 0.275 | -0.07 | 0.680 | 0.19 | 0.214 | 0.08 | 0.449 | -0.09 | 0.388 | 0.03 | 0.791 | -0.02 | 0.882 |
| **“2-back” load** | | | |  |  |  |  |  |  |  |  |  |  |  |  |  |  |  |  |  |  |  |  |  |  |  |  |
| 159 | VN | 0.07 | 0.550 | -0.16 | 0.151 | -0.04 | 0.735 | -0.22 | 0.182 | -0.02 | 0.874 | -0.15 | 0.258 | -0.12 | 0.491 | -0.14 | 0.385 | 0.18 | 0.255 | 0.17 | 0.081 | -0.02 | 0.846 | 0.10 | 0.312 | 0.01 | 0.926 |
| 160 | VN | 0.06 | 0.664 | 0.03 | 0.843 | -0.16 | 0.151 | -0.09 | 0.654 | 0.09 | 0.639 | -0.06 | 0.698 | -0.22 | 0.151 | -0.14 | 0.371 | 0.26 | 0.081 | 0.03 | 0.778 | -0.05 | 0.664 | 0.04 | 0.778 | -0.01 | 0.883 |
| 169 | VN | 0.08 | 0.510 | 0.00 | 0.956 | -0.19 | 0.081 | -0.21 | 0.188 | -0.04 | 0.832 | -0.11 | 0.466 | -0.29 | 0.054 | -0.28 | 0.061 | 0.39 | 0.010 | 0.20 | 0.036 | -0.03 | 0.778 | 0.13 | 0.185 | 0.06 | 0.609 |
| 179 | FPN | 0.02 | 0.874 | -0.01 | 0.893 | 0.11 | 0.322 | -0.10 | 0.567 | -0.16 | 0.352 | -0.20 | 0.143 | 0.02 | 0.907 | -0.03 | 0.874 | 0.18 | 0.255 | 0.21 | 0.036 | -0.02 | 0.846 | 0.07 | 0.540 | -0.06 | 0.643 |

Note: ROI refers to the index number of the node in the Power Atlas ^[29]^.

Abbreviations: FDR, False Discovery Rate correction; CPZ, chlorpromazine equivalent dose ^[30]^; FLU, fluoxetine equivalents dose ^[31]^; SANS, Scale for the Assessment of Negative Symptoms; SAPS, Scale for the Assessment of Positive Symptoms; BPRS, Brief Psychiatric Rating Scale; HAMD, Hamilton Depression Rating Scale; HAMA, Hamilton Anxiety Rating Scale; YMRS, Young Mania Rating Scale; ACC, accuracy; RT, response time; MRN, memory retrieval network; VN, visual network; FPN, frontoparietal Network.

**Table S3. Clinical and cognitive correlation of nodes with abnormal modal controllability under the “2-back” load**

| **ROI No.** | **Network** | **Illness duration** | | **CPZ** | | **FLU** | | **SAPS** | | **SANS** | | **BPRS** | | **HAMD** | | **HAMA** | | **YMRS** | | **0-back ACC** | | **0-back RT** | | **2-back ACC** | | **2-back RT** | |
| --- | --- | --- | --- | --- | --- | --- | --- | --- | --- | --- | --- | --- | --- | --- | --- | --- | --- | --- | --- | --- | --- | --- | --- | --- | --- | --- | --- |
|  |  | *r* | *p_FDR_* | *r* | *p_FDR_* | *r* | *p_FDR_* | *r* | *p_FDR_* | *r* | *p_FDR_* | *r* | *p_FDR_* | *r* | *p_FDR_* | *r* | *p_FDR_* | *r* | *p_FDR_* | *r* | *p_FDR_* | *r* | *p_FDR_* | *r* | *p_FDR_* | *r* | *p_FDR_* |
| 20 | SMN | -0.05 | 0.675 | -0.11 | 0.316 | 0.02 | 0.846 | -0.23 | 0.151 | -0.03 | 0.872 | -0.16 | 0.220 | -0.12 | 0.466 | -0.20 | 0.188 | 0.20 | 0.202 | 0.20 | 0.036 | -0.13 | 0.188 | 0.17 | 0.079 | 0.01 | 0.898 |
| 33 | SMN | 0.12 | 0.257 | 0.02 | 0.860 | 0.08 | 0.524 | -0.09 | 0.649 | 0.11 | 0.550 | -0.11 | 0.466 | -0.07 | 0.720 | -0.03 | 0.874 | 0.05 | 0.778 | 0.20 | 0.036 | -0.11 | 0.285 | 0.13 | 0.180 | -0.02 | 0.874 |
| 54 | CON | -0.02 | 0.882 | -0.08 | 0.529 | -0.02 | 0.849 | -0.28 | 0.079 | -0.11 | 0.540 | -0.23 | 0.081 | -0.11 | 0.538 | -0.07 | 0.720 | 0.32 | 0.036 | 0.23 | 0.027 | -0.09 | 0.361 | 0.08 | 0.439 | -0.02 | 0.868 |
| 69 | AN | -0.08 | 0.524 | -0.06 | 0.664 | -0.05 | 0.720 | -0.08 | 0.681 | 0.01 | 0.940 | -0.15 | 0.268 | -0.13 | 0.424 | -0.14 | 0.381 | 0.16 | 0.292 | 0.23 | 0.022 | -0.06 | 0.559 | 0.09 | 0.400 | 0.04 | 0.742 |
| 96 | DMN | 0.02 | 0.874 | -0.05 | 0.669 | 0.02 | 0.846 | -0.18 | 0.255 | 0.02 | 0.882 | -0.11 | 0.425 | -0.27 | 0.079 | -0.28 | 0.059 | 0.26 | 0.081 | 0.11 | 0.257 | -0.04 | 0.771 | 0.09 | 0.352 | -0.10 | 0.338 |
| 137 | DMN | 0.17 | 0.114 | -0.03 | 0.808 | 0.02 | 0.868 | -0.22 | 0.173 | -0.08 | 0.664 | -0.18 | 0.188 | -0.25 | 0.094 | -0.19 | 0.207 | 0.16 | 0.316 | 0.11 | 0.255 | -0.02 | 0.849 | 0.04 | 0.730 | -0.08 | 0.445 |
| 172 | VN | -0.09 | 0.466 | -0.06 | 0.639 | 0.10 | 0.392 | 0.12 | 0.524 | 0.02 | 0.883 | -0.06 | 0.707 | 0.13 | 0.404 | 0.07 | 0.700 | 0.05 | 0.778 | 0.09 | 0.376 | -0.04 | 0.766 | 0.08 | 0.439 | -0.15 | 0.151 |
| 176 | FPN | -0.03 | 0.832 | -0.04 | 0.752 | -0.12 | 0.257 | -0.10 | 0.591 | 0.05 | 0.818 | -0.08 | 0.614 | -0.10 | 0.559 | -0.06 | 0.729 | 0.25 | 0.094 | 0.18 | 0.058 | -0.06 | 0.602 | 0.06 | 0.587 | -0.03 | 0.788 |
| 187 | FPN | -0.03 | 0.800 | -0.12 | 0.271 | -0.02 | 0.874 | -0.14 | 0.404 | 0.04 | 0.842 | -0.04 | 0.832 | -0.14 | 0.389 | -0.04 | 0.832 | 0.24 | 0.114 | 0.19 | 0.047 | -0.10 | 0.334 | 0.05 | 0.662 | -0.06 | 0.593 |
| 190 | FPN | 0.06 | 0.664 | -0.02 | 0.857 | -0.12 | 0.292 | -0.09 | 0.619 | 0.12 | 0.498 | -0.10 | 0.524 | 0.03 | 0.866 | -0.07 | 0.678 | 0.16 | 0.316 | 0.03 | 0.823 | -0.07 | 0.498 | 0.09 | 0.392 | -0.08 | 0.445 |
| 191 | FPN | -0.01 | 0.926 | -0.04 | 0.778 | -0.07 | 0.567 | -0.21 | 0.188 | -0.06 | 0.778 | -0.18 | 0.180 | -0.09 | 0.609 | -0.10 | 0.587 | 0.25 | 0.090 | 0.18 | 0.073 | -0.13 | 0.188 | 0.09 | 0.392 | -0.14 | 0.180 |
| 192 | FPN | 0.03 | 0.845 | -0.06 | 0.655 | -0.07 | 0.549 | -0.18 | 0.268 | 0.04 | 0.833 | -0.13 | 0.351 | -0.14 | 0.376 | -0.11 | 0.538 | 0.25 | 0.094 | 0.04 | 0.700 | -0.09 | 0.392 | 0.03 | 0.800 | -0.14 | 0.160 |
| 194 | FPN | -0.05 | 0.678 | 0.01 | 0.934 | -0.04 | 0.730 | 0.10 | 0.587 | 0.03 | 0.868 | -0.01 | 0.929 | -0.13 | 0.444 | -0.13 | 0.445 | 0.25 | 0.090 | 0.04 | 0.757 | -0.12 | 0.207 | 0.05 | 0.664 | -0.16 | 0.114 |
| 195 | FPN | 0.02 | 0.874 | -0.06 | 0.636 | -0.06 | 0.609 | -0.17 | 0.312 | 0.03 | 0.873 | -0.15 | 0.262 | -0.20 | 0.188 | -0.14 | 0.392 | 0.23 | 0.133 | 0.10 | 0.320 | -0.13 | 0.188 | 0.02 | 0.874 | -0.04 | 0.720 |
| 197 | FPN | -0.01 | 0.902 | -0.04 | 0.778 | -0.02 | 0.874 | -0.21 | 0.188 | -0.03 | 0.874 | -0.06 | 0.676 | -0.19 | 0.220 | -0.03 | 0.874 | 0.21 | 0.180 | 0.15 | 0.121 | -0.16 | 0.094 | 0.08 | 0.471 | -0.04 | 0.778 |
| 198 | FPN | 0.06 | 0.664 | -0.13 | 0.246 | 0.06 | 0.654 | -0.10 | 0.587 | -0.02 | 0.902 | -0.09 | 0.559 | -0.20 | 0.188 | -0.13 | 0.439 | 0.17 | 0.264 | 0.17 | 0.081 | -0.18 | 0.061 | 0.11 | 0.255 | -0.05 | 0.699 |
| 199 | FPN | -0.02 | 0.874 | 0.00 | 0.955 | -0.07 | 0.558 | -0.04 | 0.846 | 0.05 | 0.778 | -0.07 | 0.655 | -0.08 | 0.664 | -0.02 | 0.902 | 0.24 | 0.104 | 0.11 | 0.262 | -0.03 | 0.832 | 0.07 | 0.491 | -0.14 | 0.180 |
| 201 | FPN | 0.06 | 0.664 | -0.12 | 0.275 | -0.02 | 0.846 | -0.14 | 0.392 | 0.01 | 0.929 | 0.02 | 0.884 | -0.15 | 0.352 | -0.09 | 0.636 | 0.18 | 0.255 | 0.13 | 0.180 | -0.16 | 0.093 | 0.10 | 0.334 | -0.02 | 0.874 |
| 202 | FPN | 0.06 | 0.639 | -0.07 | 0.549 | -0.05 | 0.698 | -0.13 | 0.466 | -0.01 | 0.940 | -0.03 | 0.846 | -0.06 | 0.729 | -0.06 | 0.730 | 0.25 | 0.094 | 0.20 | 0.036 | -0.14 | 0.143 | 0.11 | 0.271 | 0.04 | 0.735 |
| 208 | SN | 0.06 | 0.664 | 0.00 | 0.955 | -0.02 | 0.874 | -0.08 | 0.670 | -0.10 | 0.602 | -0.13 | 0.370 | -0.08 | 0.669 | -0.19 | 0.220 | 0.11 | 0.543 | 0.16 | 0.104 | -0.12 | 0.207 | 0.12 | 0.220 | -0.02 | 0.846 |
| 209 | SN | 0.04 | 0.778 | 0.03 | 0.832 | -0.03 | 0.794 | -0.18 | 0.257 | -0.13 | 0.466 | -0.21 | 0.114 | -0.04 | 0.846 | -0.02 | 0.877 | 0.19 | 0.220 | 0.18 | 0.061 | -0.01 | 0.907 | 0.05 | 0.664 | -0.12 | 0.243 |
| 213 | SN | -0.03 | 0.846 | -0.07 | 0.559 | -0.05 | 0.664 | -0.21 | 0.188 | -0.05 | 0.820 | -0.11 | 0.430 | -0.08 | 0.654 | -0.02 | 0.884 | 0.22 | 0.151 | 0.20 | 0.036 | -0.07 | 0.524 | 0.05 | 0.669 | 0.04 | 0.758 |
| 246 | Cerebellar | 0.00 | 0.964 | -0.11 | 0.322 | -0.03 | 0.800 | 0.13 | 0.466 | 0.16 | 0.318 | 0.01 | 0.955 | -0.06 | 0.730 | 0.01 | 0.934 | 0.15 | 0.355 | 0.12 | 0.246 | -0.03 | 0.778 | 0.15 | 0.130 | -0.03 | 0.801 |
| 261 | DAN | 0.03 | 0.842 | -0.07 | 0.567 | -0.03 | 0.832 | -0.13 | 0.459 | 0.05 | 0.778 | 0.01 | 0.956 | -0.02 | 0.878 | 0.09 | 0.639 | 0.18 | 0.253 | 0.19 | 0.054 | -0.13 | 0.188 | 0.02 | 0.874 | 0.00 | 0.955 |
| 182 | Uncertain | 0.07 | 0.574 | 0.06 | 0.639 | -0.13 | 0.221 | -0.10 | 0.591 | -0.03 | 0.874 | -0.09 | 0.540 | 0.03 | 0.849 | -0.12 | 0.466 | 0.26 | 0.081 | 0.21 | 0.036 | -0.12 | 0.207 | 0.16 | 0.094 | -0.10 | 0.320 |
| 183 | Uncertain | 0.09 | 0.466 | -0.04 | 0.778 | 0.03 | 0.812 | 0.16 | 0.322 | -0.06 | 0.749 | -0.04 | 0.812 | 0.00 | 0.986 | 0.04 | 0.844 | 0.13 | 0.425 | 0.20 | 0.036 | -0.16 | 0.094 | 0.12 | 0.226 | -0.07 | 0.567 |

Note: ROI refers to the index number of the node in the Power Atlas ^[29]^.

Abbreviations: FDR, False Discovery Rate correction; CPZ, chlorpromazine equivalent dose ^[30]^; FLU, fluoxetine equivalents dose ^[31]^; SANS, Scale for the Assessment of Negative Symptoms; SAPS, Scale for the Assessment of Positive Symptoms; BPRS, Brief Psychiatric Rating Scale; HAMD, Hamilton Depression Rating Scale; HAMA, Hamilton Anxiety Rating Scale; YMRS, Young Mania Rating Scale; ACC, accuracy; RT, response time; SMN, sensory/somatomotor network; CON, cingulo-opercular network; AN, auditory network; DMN, default mode network; VN, visual network; FPN, frontoparietal network; SN, salience network; DAN, dorsal attention network.

**Table S4.** **Spatial correlation between neurotransmitter and abnormal average and modal controllability**

| Receptor/transporter ^a^ | Neurotransmitter | Average controllability | | Modal controllability | |
| --- | --- | --- | --- | --- | --- |
|  |  | *r* | *p_FDR_* | *r* | *p_FDR_* |
| 5-HTR1a_1 | serotonin | -0.01 | 0.909 | 0.22 | 0.069 |
| 5-HTR1a_2 | serotonin | -0.04 | 0.885 | 0.10 | 0.338 |
| 5-HTR1b_1 | serotonin | -0.03 | 0.909 | 0.28 | 0.015 |
| 5-HTR1b_2 | serotonin | 0.01 | 0.909 | 0.05 | 0.628 |
| 5-HTR2a_1 | serotonin | 0.14 | 0.443 | 0.27 | 0.015 |
| 5-HTR2a_2 | serotonin | 0.12 | 0.443 | 0.19 | 0.125 |
| 5-HTR4 | serotonin | -0.07 | 0.669 | -0.13 | 0.254 |
| SERT_1 | serotonin | -0.15 | 0.443 | -0.10 | 0.350 |
| SERT_2 | serotonin | -0.12 | 0.443 | -0.11 | 0.335 |
| SERT_3 | serotonin | -0.12 | 0.443 | -0.14 | 0.227 |
| CBR1 | cannabinoid | -0.06 | 0.728 | 0.33 | 0.005 |
| DR1 | dopamine | -0.09 | 0.561 | -0.08 | 0.447 |
| DR2_1 | dopamine | -0.07 | 0.669 | -0.13 | 0.254 |
| DR2_2 | dopamine | -0.09 | 0.561 | -0.13 | 0.254 |
| DAT | dopamine | -0.12 | 0.443 | -0.14 | 0.227 |
| FDOPAR | fluorodopa | -0.09 | 0.561 | -0.16 | 0.179 |
| GABAaR_1 | GABA | 0.18 | 0.307 | 0.19 | 0.115 |
| GABAaR_2 | GABA | 0.23 | 0.136 | -0.05 | 0.628 |
| KOR | opioid | -0.20 | 0.262 | 0.35 | 0.005 |
| MOR_1 | opioid | -0.31 | 0.013 | 0.11 | 0.335 |
| MOR_2 | opioid | -0.33 | 0.012 | 0.14 | 0.237 |
| NAT | noradrenaline | -0.03 | 0.909 | 0.00 | 0.965 |
| NMDAR | glutamate | -0.02 | 0.909 | -0.01 | 0.913 |
| VAChT_1 | acetylcholine | -0.14 | 0.443 | -0.14 | 0.234 |
| VAChT_2 | acetylcholine | -0.14 | 0.443 | -0.16 | 0.179 |
| VAChT_3 | acetylcholine | -0.12 | 0.443 | -0.17 | 0.175 |
| mGluR5_1 | glutamate | 0.08 | 0.669 | 0.24 | 0.036 |
| mGluR5_2 | glutamate | 0.01 | 0.909 | 0.27 | 0.015 |
| mGluR5_3 | glutamate | -0.02 | 0.909 | 0.28 | 0.015 |

Note: ^a^ Neurotransmitter receptors/transporters with suffixes _1, _2 indicate different tracers ^[63]^.

Abbreviations: FDR, False Discovery Rate correction; 5-HTR, 5-hydroxytryptamine receptor; SERT, serotonin transporter; CBR, cannabinoid receptor; DR, dopamine receptor; DAT, dopamine transporter; FDOPAR, fluorodopa receptor; GABAaR, gamma-aminobutyric acid a receptor; KOR, kappa opioid receptor; MOR, mu opioid receptor; NAT, noradrenaline transporter; NMDAR, N-methyl-D-aspartic acid receptor; VAChT, vesicular acetylcholine transporter; mGluR5, metabotropic glutamate type 5.

**Table S5. Spatial correlation between neurotransmitter and SZ-specific abnormal average and modal controllability under “0-back” and “2-back” loads**

| Receptor/transporter ^a^ | Neurotransmitter | 0-back Average controllability | | 2-back Average controllability | | 2-back Modal  controllability | |
| --- | --- | --- | --- | --- | --- | --- | --- |
|  |  | *r* | *p_FDR_* | *r* | *p_FDR_* | *r* | *p_FDR_* |
| 5-HTR1a_1 | serotonin | -0.02 | 0.942 | 0.04 | 0.847 | 0.16 | 0.172 |
| 5-HTR1a_2 | serotonin | -0.06 | 0.942 | 0.01 | 0.960 | 0.08 | 0.472 |
| 5-HTR1b_1 | serotonin | 0.01 | 0.942 | -0.06 | 0.813 | 0.22 | 0.078 |
| 5-HTR1b_2 | serotonin | -0.03 | 0.942 | 0.00 | 0.979 | 0.02 | 0.845 |
| 5-HTR2a_1 | serotonin | 0.04 | 0.942 | 0.10 | 0.813 | 0.23 | 0.064 |
| 5-HTR2a_2 | serotonin | 0.01 | 0.942 | 0.09 | 0.813 | 0.17 | 0.172 |
| 5-HTR4 | serotonin | -0.02 | 0.942 | -0.04 | 0.847 | -0.13 | 0.264 |
| SERT_1 | serotonin | 0.03 | 0.942 | -0.06 | 0.813 | 0.27 | 0.036 |
| SERT_2 | serotonin | 0.01 | 0.942 | -0.06 | 0.813 | -0.10 | 0.370 |
| SERT_3 | serotonin | -0.02 | 0.942 | -0.06 | 0.813 | -0.12 | 0.267 |
| CBR1 | cannabinoid | -0.04 | 0.942 | -0.05 | 0.813 | -0.14 | 0.237 |
| DR1 | dopamine | -0.02 | 0.942 | -0.08 | 0.813 | -0.16 | 0.172 |
| DR2_1 | dopamine | -0.02 | 0.942 | -0.05 | 0.813 | -0.16 | 0.172 |
| DR2_2 | dopamine | 0.01 | 0.942 | 0.10 | 0.813 | 0.17 | 0.172 |
| DAT | dopamine | -0.02 | 0.942 | 0.10 | 0.813 | -0.02 | 0.845 |
| FDOPAR | fluorodopa | -0.08 | 0.942 | -0.13 | 0.813 | 0.27 | 0.036 |
| GABAaR_1 | GABA | -0.06 | 0.942 | -0.17 | 0.813 | 0.08 | 0.472 |
| GABAaR_2 | GABA | -0.06 | 0.942 | -0.17 | 0.813 | 0.09 | 0.386 |
| KOR | opioid | -0.03 | 0.942 | 0.04 | 0.847 | -0.04 | 0.699 |
| MOR_1 | opioid | 0.04 | 0.942 | -0.03 | 0.867 | -0.07 | 0.538 |
| MOR_2 | opioid | -0.01 | 0.942 | -0.11 | 0.813 | -0.12 | 0.264 |
| NAT | noradrenaline | -0.02 | 0.942 | -0.09 | 0.813 | -0.12 | 0.264 |
| NMDAR | glutamate | -0.02 | 0.942 | -0.07 | 0.813 | -0.16 | 0.172 |
| VAChT_1 | acetylcholine | -0.04 | 0.942 | -0.09 | 0.813 | -0.13 | 0.247 |
| VAChT_2 | acetylcholine | -0.03 | 0.942 | -0.11 | 0.813 | -0.17 | 0.172 |
| VAChT_3 | acetylcholine | -0.04 | 0.942 | -0.09 | 0.813 | -0.17 | 0.172 |
| mGluR5_1 | glutamate | 0.02 | 0.942 | 0.05 | 0.813 | 0.20 | 0.119 |
| mGluR5_2 | glutamate | 0.01 | 0.942 | 0.03 | 0.869 | 0.24 | 0.064 |
| mGluR5_3 | glutamate | 0.03 | 0.942 | 0.00 | 0.979 | 0.26 | 0.048 |

Note: ^a^ Neurotransmitter receptors/transporters with suffixes _1, _2 indicate different tracers ^[63]^.

Abbreviations: FDR, False Discovery Rate correction; 5-HTR, 5-hydroxytryptamine receptor; SERT, serotonin transporter; CBR, cannabinoid receptor; DR, dopamine receptor; DAT, dopamine transporter; FDOPAR, fluorodopa receptor; GABAaR, gamma-aminobutyric acid a receptor; KOR, kappa opioid receptor; MOR, mu opioid receptor; NAT, noradrenaline transporter; NMDAR, N-methyl-D-aspartic acid receptor; VAChT, vesicular acetylcholine transporter; mGluR5, metabotropic glutamate type 5.

**Table S6. Spatial correlation between neurotransmitter and transdiagnostic abnormal average and modal controllability under the “2-back” load**

| Receptor/ transporter  ^a^ | Neurotransmitter | SZ and MDD Average controllability | | SZ and MDD Modal controllability | | SZ and BD Modal controllability | | SZ, BD, and MDD Modal controllability | |
| --- | --- | --- | --- | --- | --- | --- | --- | --- | --- |
|  |  | *r* | *p_FDR_* | *r* | *p_FDR_* | *r* | *p_FDR_* | *r* | *p_FDR_* |
| 5-HTR1a_1 | serotonin | -0.05 | 0.767 | 0.14 | 0.440 | 0.02 | 0.880 | 0.09 | 0.950 |
| 5-HTR1a_2 | serotonin | -0.06 | 0.733 | 0.05 | 0.789 | 0.00 | 0.958 | 0.05 | 0.950 |
| 5-HTR1b_1 | serotonin | 0.01 | 0.924 | 0.12 | 0.695 | 0.10 | 0.752 | 0.03 | 0.950 |
| 5-HTR1b_2 | serotonin | 0.02 | 0.914 | 0.04 | 0.809 | -0.07 | 0.772 | 0.00 | 0.985 |
| 5-HTR2a_1 | serotonin | 0.10 | 0.682 | 0.17 | 0.330 | 0.09 | 0.752 | 0.12 | 0.950 |
| 5-HTR2a_2 | serotonin | 0.08 | 0.682 | 0.09 | 0.789 | 0.03 | 0.880 | 0.09 | 0.950 |
| 5-HTR4 | serotonin | -0.06 | 0.733 | -0.05 | 0.789 | -0.09 | 0.752 | -0.02 | 0.950 |
| SERT_1 | serotonin | -0.04 | 0.805 | 0.21 | 0.330 | 0.13 | 0.752 | 0.06 | 0.950 |
| SERT_2 | serotonin | -0.08 | 0.682 | -0.05 | 0.789 | -0.08 | 0.752 | -0.06 | 0.950 |
| SERT_3 | serotonin | -0.04 | 0.795 | -0.05 | 0.789 | -0.06 | 0.841 | -0.05 | 0.950 |
| CBR1 | cannabinoid | -0.07 | 0.682 | -0.03 | 0.816 | -0.08 | 0.752 | -0.05 | 0.950 |
| DR1 | dopamine | -0.09 | 0.682 | -0.05 | 0.789 | -0.14 | 0.752 | -0.09 | 0.950 |
| DR2_1 | dopamine | -0.08 | 0.682 | -0.08 | 0.789 | -0.14 | 0.752 | -0.07 | 0.950 |
| DR2_2 | dopamine | 0.16 | 0.679 | 0.15 | 0.440 | 0.03 | 0.880 | 0.04 | 0.950 |
| DAT | dopamine | 0.23 | 0.135 | 0.06 | 0.789 | -0.02 | 0.880 | 0.02 | 0.950 |
| FDOPAR | fluorodopa | -0.14 | 0.682 | 0.22 | 0.330 | 0.02 | 0.880 | -0.02 | 0.950 |
| GABAaR_1 | GABA | -0.27 | 0.065 | -0.01 | 0.885 | 0.04 | 0.880 | 0.00 | 0.985 |
| GABAaR_2 | GABA | -0.29 | 0.065 | 0.02 | 0.833 | 0.02 | 0.880 | 0.00 | 0.985 |
| KOR | opioid | -0.08 | 0.682 | 0.11 | 0.744 | -0.08 | 0.752 | 0.03 | 0.950 |
| MOR_1 | opioid | -0.01 | 0.924 | 0.06 | 0.789 | -0.12 | 0.752 | -0.11 | 0.950 |
| MOR_2 | opioid | -0.11 | 0.682 | -0.04 | 0.809 | -0.14 | 0.752 | -0.11 | 0.950 |
| NAT | noradrenaline | -0.08 | 0.682 | -0.03 | 0.816 | -0.10 | 0.752 | -0.09 | 0.950 |
| NMDAR | glutamate | -0.09 | 0.682 | -0.06 | 0.789 | -0.14 | 0.752 | -0.08 | 0.950 |
| VAChT_1 | acetylcholine | -0.10 | 0.682 | -0.07 | 0.789 | -0.05 | 0.872 | -0.08 | 0.950 |
| VAChT_2 | acetylcholine | -0.09 | 0.682 | -0.06 | 0.789 | -0.10 | 0.752 | -0.10 | 0.950 |
| VAChT_3 | acetylcholine | -0.09 | 0.682 | -0.07 | 0.789 | -0.08 | 0.752 | -0.08 | 0.950 |
| mGluR5_1 | glutamate | 0.05 | 0.767 | 0.19 | 0.330 | 0.08 | 0.752 | 0.07 | 0.950 |
| mGluR5_2 | glutamate | -0.01 | 0.924 | 0.17 | 0.330 | 0.04 | 0.880 | 0.05 | 0.950 |
| mGluR5_3 | glutamate | -0.04 | 0.805 | 0.19 | 0.330 | 0.01 | 0.947 | 0.01 | 0.985 |

Note: ^a^ Neurotransmitter receptors/transporters with suffixes _1, _2 indicate different tracers ^[63]^.

Abbreviations: FDR, False Discovery Rate correction; 5-HTR, 5-hydroxytryptamine receptor; SERT, serotonin transporter; CBR, cannabinoid receptor; DR, dopamine receptor; DAT, dopamine transporter; FDOPAR, fluorodopa receptor; GABAaR, gamma-aminobutyric acid a receptor; KOR, kappa opioid receptor; MOR, mu opioid receptor; NAT, noradrenaline transporter; NMDAR, N-methyl-D-aspartic acid receptor; VAChT, vesicular acetylcholine transporter; mGluR5, metabotropic glutamate type 5.
